# CRISPR‐Knockout Screen Identifies Dmap1 as a Regulator of Chemically Induced Reprogramming and Differentiation of Cardiac Progenitors

**DOI:** 10.1002/stem.3012

**Published:** 2019-04-23

**Authors:** Jason S. L. Yu, Giorgia Palano, Cindy Lim, Aldo Moggio, Lauren Drowley, Alleyn T. Plowright, Mohammad Bohlooly‐Y, Barry S. Rosen, Emil M. Hansson, Qing‐Dong Wang, Kosuke Yusa

**Affiliations:** ^1^ Stem Cell Genetics, Wellcome Sanger Institute Hinxton, Cambridge United Kingdom; ^2^ KI/AZ Integrated CardioMetabolic Center (ICMC), Department of Medicine Karolinska Institutet Huddinge Sweden; ^3^ Bioscience Heart Failure, Cardiovascular, Renal and Metabolism, IMED Biotech Unit AstraZeneca Gothenburg Sweden; ^4^ Medicinal Chemistry, Cardiovascular, Renal and Metabolism, IMED Biotech Unit AstraZeneca Gothenburg Sweden; ^5^ Discovery Sciences, IMED Biotech Unit AstraZeneca Gothenburg Sweden; ^6^ Department of Cell Biology The Francis Crick Institute London United Kingdom; ^7^ Stem Cell Genetics Institute for Frontier Life and Medical Sciences, Kyoto University Kyoto Japan

**Keywords:** Cardiac progenitors, Chemical reprogramming, Clustered regularly interspaced short palindromic repeats‐Cas9, Genome‐wide screen, CpG methylation

## Abstract

Direct in vivo reprogramming of cardiac fibroblasts into myocytes is an attractive therapeutic intervention in resolving myogenic deterioration. Current transgene‐dependent approaches can restore cardiac function, but dependence on retroviral delivery and persistent retention of transgenic sequences are significant therapeutic hurdles. Chemical reprogramming has been established as a legitimate method to generate functional cell types, including those of the cardiac lineage. Here, we have extended this approach to generate progenitor cells that can differentiate into endothelial cells and cardiomyocytes using a single inhibitor protocol. Depletion of terminally differentiated cells and enrichment for proliferative cells result in a second expandable progenitor population that can robustly give rise to myofibroblasts and smooth muscle. Deployment of a genome‐wide knockout screen with clustered regularly interspaced short palindromic repeats‐guide RNA library to identify novel mediators that regulate the reprogramming revealed the involvement of DNA methyltransferase 1‐associated protein 1 (Dmap1). Loss of Dmap1 reduced promoter methylation, increased the expression of Nkx2‐5, and enhanced the retention of self‐renewal, although further differentiation is inhibited because of the sustained expression of Cdh1. Our results hence establish Dmap1 as a modulator of cardiac reprogramming and myocytic induction. stem cells *2019;37:958–972*

**Significance Statement**

The present study demonstrates the chemically induced conversion of mouse cardiac fibroblasts into progenitors using transforming growth factor‐β/Alk5 inhibition coupled with hypoxia. Utilizing this protocol, two progenitor populations of differing potency were generated: an initial population that could give rise to endothelial and cardiomyocyte lineages and a second population that is more lineage restricted toward myofibroblast and smooth muscle. In characterizing the biology behind this reprogramming using a clustered regularly interspaced short palindromic repeats‐knockout‐based genome‐wide screen, this study aims to drive the development of novel therapeutics that can improve cardiac function by promoting the in situ induction of cardiac progenitors.

## Introduction

Cardiovascular disease (CVD) represents the single leading cause of mortality throughout the world 1. Myocardial infarction is the most common consequence of CVD, whereby acute coronary thrombosis leads to large‐scale cardiomyocyte (CM) death and fibrotic scarring. Fibrotic repair is inadequate reparation for CM loss, weakening the contractile properties of the heart and increasing the risk of heart failure and mortality. Although the mammalian heart has some, albeit limited capacity for cardiomyogenesis, this largely restricted to early developmental stages 2, 3, 4 and is insufficient in replacing the large amounts of CM lost following infarction. Transplantation of pluripotent stem cells (PSCs)‐derived CM has been shown to improve cardiac function in primate models, but low engraftment efficiency coupled with the sheer number required to restore cardiac function represents significant therapeutic hurdles 5, 6. Consequently, there is a dire need to derive cardiac cell types that are more amenable for regenerative therapies.

Analogous to many other organs, cardiac progenitors (CPs) have been identified in the rodent and human adult heart, which upon isolation and transplantation have varying levels of success in ameliorating cardiac dysfunction 7, 8, 9, 10, 11. However, there is currently a lack of consensus as to the exact molecular identity that define CP 12. Moreover, these cells have either no or limited cardiomyogenic potential 13, 14, and their beneficial effects upon the heart post‐transplantation is likely because of paracrine effects rather than true restoration of CM 15. Furthermore, their relative scarcity and difficulty of isolation precludes any meaningful use as regenerative therapy. In contrast, Nkx2‐5^+^ CPs, which emerge during embryogenesis, are bipotent and can differentiate into CM and smooth muscle (SM), thus possessing a greater regenerative capacity and serving as a useful marker of progenitor properties 16. However, these cells are unable to sustain these properties in vitro and are unsuitable for direct therapeutic use. Nonetheless, direct reprogramming approaches converting terminally differentiated mouse cardiac fibroblast (CF) into multipotent CP by transient expression of cardiogenic factors and chemically defined expansion have been reported 17, 18. However, such approaches ultimately depend on exogenous gene expression and genomic integration of transgenes, which compromises their use in a therapeutic setting.

Fully chemically induced approaches have the potential to generate efficacious progenitor populations that are more suited for therapeutic use. Small molecules are more readily amenable to drug development, potentiating the in situ regeneration of the heart and other organs. Chemical reprogramming has already permitted the derivation of induced PSCs, hepatocytes, neurons, and CM from mouse and human fibroblasts, utilizing a variety of chemical compounds that act to alter the epigenetic and gene expression networks to redirect cells to a new fate 19, 20, 21, 22. Although this approach is capable of generating terminally differentiated cell types in vitro, whether these cells are truly fully functional remains unknown. Furthermore, it is also unclear whether such an approach can generate progenitor populations that retain differentiation potency concurrent with self‐renewal, as the genetic and epigenetic pathways regulating these processes are undefined.

Murine CF demonstrates remarkable plasticity as forced expression of cardiogenic factors *Gata4, Mef2c*, and *Tbx5* induces in vivo conversion into CM 23. Suppression of fibrotic signaling via small molecules improves the kinetics of this reprogramming, whereas targeted suppression of transforming growth factor‐β (TGF‐β) receptors Alk4,5,7 increases the CM yield 24, 25, establishing TGF‐β signaling as a key axis regulating conversion toward the cardiac lineage 26. Additionally, there is evidence supporting the ability of hypoxic conditions to stimulate cardiac regeneration via decreased myocardial fibrosis 27, thereby defining the conditions through which small molecules are able to induce the formation of regenerative progenitors in a transgene‐free context.

In this study, we have developed a robust chemically induced reprogramming protocol that permits the derivation of progenitor populations from mouse CF independent of transgene‐mediated approaches. Initially, the resultant progenitor colonies can spontaneously give rise to endothelial cells (ECs) and CM; however, depletion of these differentiated cells leads to the emergence of a second progenitor population that is readily expandable, although differentiation potency appears restricted to the myofibroblast and SM lineages. Consequently, we performed a genome‐wide clustered regularly interspaced short palindromic repeats (CRISPR)‐Cas9 knockout screen to uncover the factors and pathways that could potentiate this reprogramming process. Our data further extend the utility of chemical‐induced derivation of progenitor populations and constitutes a proof‐of‐principle demonstration of the power of forward genetic screens in uncovering the molecular basis behind this approach.

## Materials and Methods

### Lineage Tracing and Reporter Mouse Lines

All animal experiments conducted in the United Kingdom were carried out in accordance with the Animals (Scientific Procedures) Acts 1986 and approved by the Wellcome Sanger Institute Ethics Committee. All animal experiments conducted in Sweden were performed in accordance with Swedish legislation and approved by the Stockholm South Animal Ethics Committee and the Gothenburg Ethics Committee for Experimental Animals (permit 47/15, 91‐2013 and 95‐2014, respectively). Cas9‐expressing mouse line was generated by inserting the human *EF1a* promoter‐driven Cas9 expression cassette into the *Rosa26* locus in mouse embryonic stem cell (ESC) line JM8 and is kept in the C57BL/6N background and used for CF isolation. ROSA26R^NKX2‐5 Enhancer‐BP‐EGFP^ reporter mice were used to inform on Nkx2‐5 enhancer activity (generated in house, AstraZeneca). B6N.Cg‐Tg(Pdgfra‐cre/ERT)467Dbe/J mice (018280, Bar Harbor, Jackson Labs) were crossed with reporter mice Gt(ROSA)26Sor^tm1.1(CAG‐EGFP)Fsh^ (32037‐JAX, Jackson Labs). Recombination was induced via gavage of 2 mg tamoxifen (Sigma‐Aldrich), resuspended in corn oil (20 mg/ml, Sigma‐Aldrich), 1 day before heart isolation in 12‐week‐old mice. Immediately before sacrifice, heparin (1,000 IU, LEO Pharma) was injected intraperitoneally. The hearts were rinsed in ice‐cold Dulbecco's phosphate‐buffered saline (without Ca^2+^ and Mg^2+^, Thermo Fisher Scientific) and processed for either fixation in paraformaldehyde (PFA), sectioning, and immunofluorescence using standard protocols or CF isolation, fluorescence‐activated cell sorting (FACS) of enhanced green fluorescent protein positive (EGFP+) cells, and chemically induced reprogramming and differentiation experiments as described below. For sectioning, fixed hearts were incubated in 30% sucrose solution for 24 hours at 4°C and then embedded in OCT (Tissue‐Tek O.C.T. Compound, Sakura Finetek) for cryopreservation and cryostat sectioning. Sections (10 μm thick) were rinsed in phosphate‐buffered saline (PBS), permeabilized in PBS containing 0.3% Triton‐X100, and then incubated in 5% bovine serum albumin (BSA; Sigma‐Aldrich) solution at room temperature (RT). Primary antibodies were diluted in PBS supplemented with 0.1% BSA and Triton‐X100 and incubated overnight at 4°C. After PBS wash, fluorescently labeled secondary antibodies (Thermo Fisher Scientific) were diluted in staining buffer and incubated for 1 hour at RT. Hoechst 33342 solution (Sigma‐Aldrich) was added for nuclear staining. Images were acquired using a Zeiss Axio Observer Z1‐Axiocam 506 mono microscope (Zeiss). A list of antibodies and dilutions are available in Supporting Information Table S3.

### Cardiac Fibroblast Isolation

The hearts were isolated from 8‐ to 12 ‐week‐old Cas9^+^ mice and transferred to sterile cold Hank's balanced salt solution (HBBS; Sigma) + 2x Pen/Strep (Life Technologies). The hearts were then dissected, rinsed, and finely minced before transfer into digestion buffer prewarmed to 37°C (980 U/ml collagenase II [Life Technologies], 20 KU/ml DNase I [Stem Cell Technologies] in HBSS + 1x Pen/Strep) for 10 minutes. Digestion was then centrifuged for 5 minutes at 600*g* and incubated with 5 ml TryPLE (Life Technologies) for 5 minutes. Entire pellet was resuspended in 4.5 ml prewarmed CF media (15% fetal bovine serum [FBS], 1% NEAA, 1x Pen/Strep in Dulbecco's modified Eagle's medium [DMEM]+GlutaMAX [Life Technologies]) and passed through a 40 μm filter to remove any large undigested pieces. Cell suspension was plated onto four wells of a 0.1% gelatin‐coated six‐well plate. Plate was incubated overnight at 37°C and media was exchanged the following day. When confluent, cells were passaged once with 0.5% trypsin‐EDTA (Life Technologies) before being frozen down at 3 × 10^5^ cells per milliliter in 90% FBS + 10% dimethyl sulfoxide (DMSO; Sigma‐Aldrich).

### Chemically Induced Reprogramming and Maintenance of Progenitors

Plates were coated overnight with laminin‐211 (BioLamina, 10 μg/ml). CF were trypsinized, washed twice with PBS, and plated onto laminin‐211‐coated plates at 1140 cells per centimeter square in reprogramming media (5% Knockout™ Serum Replacement, 15% ESC qualified FBS, 1% non‐essential amino acid, and 0.1 mM β‐mercaptoethanol in DMEM+GlutMAX [Life Technologies] supplemented with 2 μM SB431542 [Selleck Chem] or RepSox/AZ12799734 [AstraZeneca] under 5% O_2_). Reprogramming was conducted for 17 days during which colonies of CP emerge and proliferate. Upon completion, individual colonies were picked and expanded under reprogramming conditions to enrich for chemically induced smooth muscle progenitor (ciSMP). Cells were passaged at least five times to ensure complete depletion of nonreprogrammed/differentiated cells. ciSMPs were routinely maintained in reprogramming media and frozen at 1 × 10^6^ cells in 90% ESC‐qualified FBS + 10% DMSO.

### Differentiation of ciSMP

Growth factor reduced Matrigel (BD Biosciences)‐coated plates were prepared at 1:30 dilution in DMEM+GlutaMAX to provide a prodifferentiation matrix. SM differentiation was initiated by passaging ciSMP onto these plates in differentiation media (2% FBS in DMEM+GlutaMAX) supplemented with 5 ng/ml TGF‐β (Cambridge Bioscience) and cultured in a humidified incubator at 37°C with 5% CO_2_ for 3–7 days with media change every 2 days.

### CRISPR‐Knockout Screening and Hit Validation

Screening was conducted as previously described 28 with modifications. In brief, upon validation of Cas9 activity using self‐targeting green fluorescent protein (GFP) reporter (Supporting Information Fig. S2A), CFs (p1) were trypsinized and transduced with mouse genome‐wide lentiviral CRISPR‐guide RNA (gRNA) library 2 (Addgene, 67,988) at a multiplicity of infection of 0.3, which was confirmed by flow cytometry for BFP expression 2 days post‐transduction. Mutant CF library (p2) was then selected with 3 μg/ml puromycin (Sigma‐Aldrich) for 4 days and then passaged into reprogramming conditions for 17 days. Following reprogramming, cells were trypsinized and subjected to nuclear extraction and immunostaining for Nkx2‐5 before FACS to isolate Nkx2‐5^LOW^ and Nkx2‐5^HIGH^ populations. Following decrosslinking and genomic DNA extraction, gRNA‐containing regions were amplified via two rounds of polymerase chain reaction (PCR), before submission for sequencing. Statistical analysis and generation of hit list was conducted via model‐based analysis of genome‐wide CRISPR‐Cas9‐knockout (MAGeCK). For validation experiments, single guides were cloned into lentiviral CRISPR backbone (pPGK‐U6gRNA[BbsI]‐Puro‐2A‐BFP, Addgene 50,946) and transduced into CF before reprogramming following the same timescale. A list of raw gRNA read‐counts and MAGeCK output is available in Supporting Information Table S1. A list of gRNA is available in Supporting Information Table S2.

### Intracellular Staining and FACS

In brief, trypsinized cells were incubated on ice for 10 minutes in hypotonic buffer (20 mM Tris‐HCl, 0.1 mM EDTA, 5 mM MgCl_2_; Sigma‐Aldrich) before the addition of 1% Triton‐X‐100 (Sigma‐Aldrich) to induce plasma membrane lysis. Solution was then centrifuged for 3 minutes at 500*g* and washed twice with hypotonic buffer +0.5% Triton‐X‐100 to further purify nuclei. Nuclear pellet was then fixed for 10 minutes at RT with 4% PFA (Thermo Fisher) with agitation. Nuclei were then washed once with cold PBS and subjected to immunostaining with Nkx2‐5 antibody (Santa Cruz) followed by FACS.

### Immunostaining

Cells were fixed in 4% PFA for 10 minutes at RT. Fixed cells were then blocked and permeabilized with the appropriate buffer (10% goat/donkey serum [Sigma‐Aldrich], 0.5% Triton‐X‐100, 2.5% IgG‐free BSA [Jackson Labs]) in PBS for 1 hour at RT. Cells were then incubated with primary antibody overnight at 4°C with agitation in blocking/permeablizing buffer without Triton‐X‐100. Next day, cells were washed thrice and incubated with PBS‐diluted secondary 1 hour at RT in darkness. Cells were then incubated for 30 minutes with 1:1,000 dilution 4′,6‐diamidino‐2‐phenylindole (DAPI; Sigma‐Aldrich) and phalloidin (PL) counterstain (Life Technologies), and imaged via confocal microscopy (LM700, Zeiss).

### Immunoblotting

Cells were lysed in RIPA buffer (Sigma‐Aldrich) supplemented with protease and phosphatase inhibitor cocktails at 1:100 dilution (Sigma‐Aldrich). Protein quantification (BCA kit) and immunoblotting (NuPage system) was performed as per manufacturer's instructions (Thermo Fisher Scientific, Life Technologies). Proteins were transferred onto polyvinylidene difluoride membranes via electroblotting following manufacturer's instructions (BioRad). Membranes were incubated overnight with primary antibody in 5% BSA/milk in tris‐buffered saline with 0.1% Tween 20, followed by 1 hour incubation at RT with the appropriate secondary antibody. Membranes were developed using enhanced chemiluminescence substrate (Thermo Fisher Scientific) onto radiographic film (Amersham). A list of antibodies is available in Supporting Information Table S3.

### Semiquantitative and Quantitative PCR

Total RNA was extracted using RNA Plus Mini Kit (Qiagen) following manufacturer's instructions. About 0.5–2 μg of RNA was used for subsequent cDNA synthesis via Superscript III (Life Technologies). For semiquantitative PCR, standard PCR was performed using Platinum Taq Polymerase High Fidelity (Life Technologies) for the appropriate number of cycles for each gene, which was determined empirically. For quantitative real‐time PCR, 3 microliter per reaction of 1:20 dilution of cDNA was used and real‐time amplification performed following manufacturer's instructions (Brilliant III Ultra‐Fast SYBR Green PCR Mix with Low ROX, Agilent). Data obtained are from three biological replicates (*n* = 3), each consisting of two technical replicates. A list of primers used is available in Supporting Information Table S4.

### Bisulfite Sequencing and Promoter Methylation Analysis

Genomic DNA was extracted and converted via bisulfite reaction following manufacturer's instructions (DNeasy Blood & Tissue kit, EpiTect Fast Bisulfite Conversion kit, Qiagen). Primers were designed to amplify CpG island regions adjacent or flanking the transcription start site (TSS) to gauge levels of promoter methylation. Regions were amplified from using 200 ng of bisulfite‐treated template and DNA polymerase (EpiMark Hot Start Taq Polymerase, NEB). Resultant PCR product was cloned into a sequencing plasmid following manufacturer's instruction (PCR Cloning Kit, NEB) and submitted for Sanger sequencing. A list of primers used is available in Supporting Information Table S5.

### Cloning and Generation of Cas9‐Resistant DNA Methyltransferase 1‐Associated Protein 1 Rescue Mutant

Primers were designed to clone out the wild‐type DNA methyltransferase 1‐associated protein 1 (Dmap1) coding sequence from cDNA using high‐fidelity PCR (Q5 Hot Start Taq Polymerase, NEB). Subsequent PCR product was cloned into lentiviral vector encoding for blastocydin resistance and BFP (EF1α‐BFP‐2A‐Bsd‐2A‐Dmap1). To ensure that Dmap1 can be encoded in the presence of Cas9 activity and g4 gRNA expression, site directed mutagenesis was performed to mutagenize the g4‐gRNA binding site while retaining the native amino acid encoding sequence (5′‐CCTTTTACTAACCCAGCTCGAAA‐3′ to 5′‐CCCTTCACCAATCCCGCCCGCAA‐3′) following manufacturer's instructions (Q5 Site Directed Mutagenesis Kit, NEB). Transgene was introduced into Dmap1‐knockout (KO) cells via lentiviral transduction. Plasmids used in this study are available from https://www.addgene.org/Kosuke_Yusa/.

## Results

### Alk5 Inhibition Efficiently Promotes the Derivation of Cardiac Progenitors

**Figure 1:**
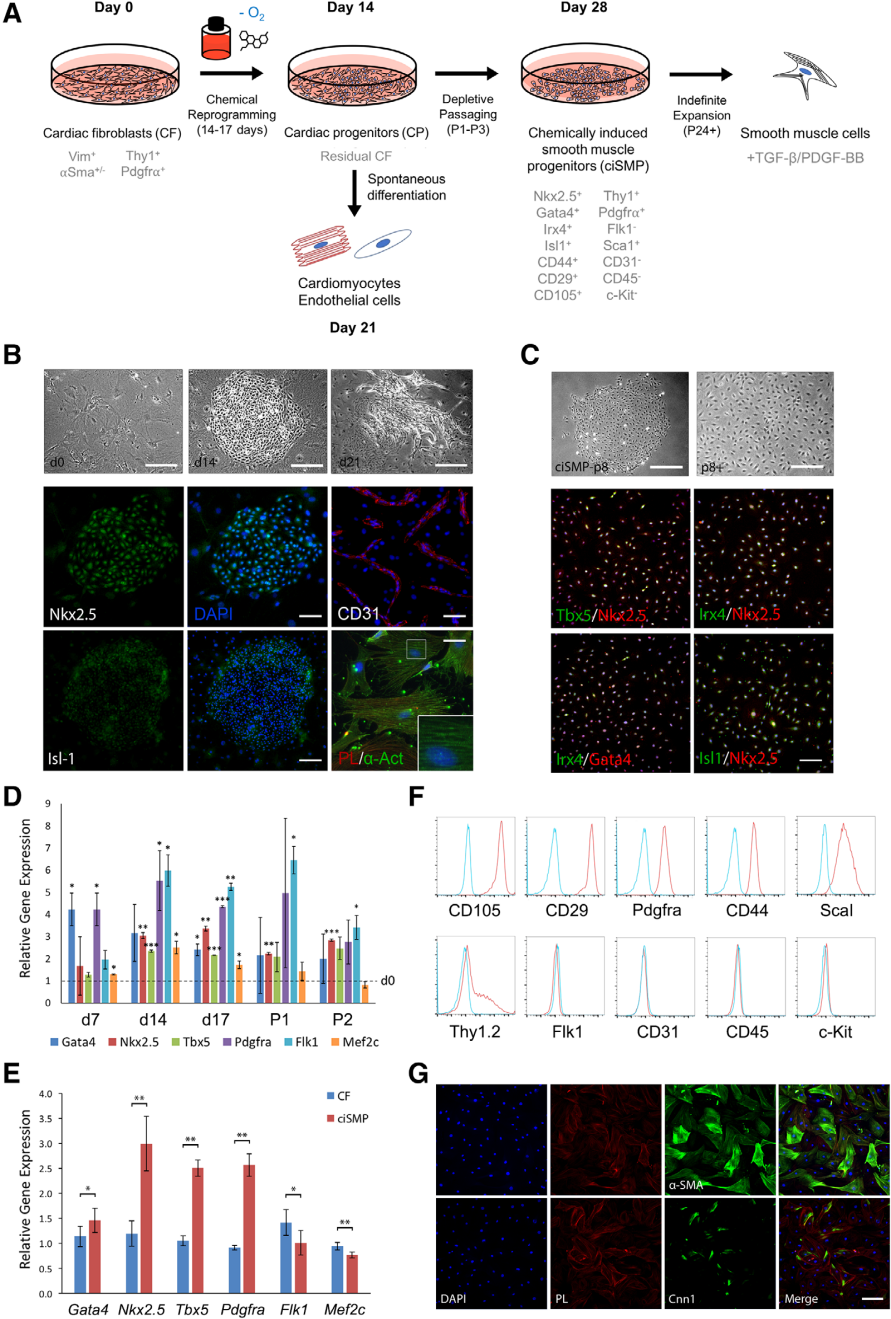
Single factor reprogramming induces formation of CP and ciSMP from CF. **(A):** Schematic depicting reprogramming protocol and markers associated with each stage. **(B):** Phase contrast of d0 CF and d14 ciSMP colonies and subsequent immunostain for Nkx2.5 and Isl‐1. Phase contrast of endothelial tubules emerging from a differentiated colony at d21 confirmed by CD31 staining. Cells from passaged d21 differentiated colonies were stained with α‐actinin, confirming the presence of cardiomyocyte. Inserts indicate magnified region showing striated myofibrils. **(C):** Phase contrast of enriched ciSMP and subsequent immunostain for indicated CP markers demonstrating homogenous coexpression. Cells are counterstained with DAPI (blue channel). **(D):** Timecourse GEA of CP markers across the reprogramming process. Dotted line indicates d0, whereby relative gene expression (RGE) = 1. Statistical significance is with respect to d0. **(E):** Gene expression analysis of CP markers relative to CF in ciSMP. Error bars indicate SD. *, **, and *** indicate *p* < .05, *p* < .01, and *p* < .001, respectively, as calculated by Student's *t* test, where *n* = 3. **(F):** Fluorescence‐activated cell sorting staining for CP surface markers in ciSMP (red) verses isotype control (blue). **(G):** Further differentiation to smooth muscle. Scale bars indicate 400 μm (Isl‐1 immunostain), 200 μm (Nkx2‐5 immunostain, d10, d14, d21 ciSMP‐p8), 100 μm (P8+, CD31 immunostain), or 50 μm (α‐actinin immunostain). See also Supporting Information Figures S1 and S2. Abbreviations: α‐SMA, alpha‐smooth muscle actin; CF, cardiac fibroblast; ciSMP, chemically induced smooth muscle progenitor; Cnn1, calponincalponin; CP, cardiac progenitor; DAPI, 4′,6‐diamidino‐2‐phenylindole; PL, phalloidin; TGF‐β, transforming growth factor‐β.

To explore the rationale by which small molecules are able to induce the derivation of the progenitor populations, we designed and implemented a robust protocol to convert CF into potential CP (Fig. 1A). Based on the importance of TGF‐β signaling and hypoxia in mediating cardiac reprogramming and organ regeneration 24, 25, 27, 29, we reasoned that TGF‐β pathway inhibition under sustained hypoxia may potentiate the reprogramming of fibroblasts into progenitor populations. As a basal matrix, we used laminin‐211 as this has been documented to support development of the heart and to drive the expansion of CP derived from mesenchymal stromal cells (MSCs) 30, 31. Upregulation of CP marker expression, particularly *Nkx2‐5*, is a key hallmark of progenitor identity during development 16, 32 and has therefore been used as the metric to assess reprogramming efficiency 16, 17, 18. Validation of the initial isolation step confirmed the purification of fibroblasts as evidenced by pervasive vimentin (VIM) staining (Supporting Information Fig. S1A). Concurrently, the majority of cells expressed Thy1.2 and PDGFRα cell surface markers which are characteristically associated with fibroblasts and subpopulations of CP (Supporting Information Fig. S1B) 11, 33. However, isolated cells also displayed heterogeneous expression of alpha‐SM actin (α‐SMA), suggesting the presence of a mixed cell population of both fibroblasts and myofibroblasts. Nonetheless, when these cells are subjected to reprogramming conditions (5% O_2_, 2 μM SB431542/Alk5i), morphological examination revealed the emergence of several highly proliferative, colony‐like structures (Fig. 1B). Immunostaining of d14 colonies showed that they were positive for Nkx2‐5 and Isl‐1 (Fig. 1B, 1C). Although hypoxic conditions alone (i.e., in the DMSO control) remained largely monolayer and generated far fewer colonies compared with Alk5i (Supporting Information Fig. S1C), when cultured up to and beyond 3 weeks under the same conditions, we observed spontaneous differentiation of some colonies into EC and CM lineages as evidenced by clear morphological changes and positive CD31 or α‐actinin staining (Fig. 1B, 1D; Supporting Information S1D). The ability of these colonies to undergo further differentiation suggests the presence of true CP populations. When d21 colonies were subsequently dissociated and replated, cells adopted characteristic CM features such as α*‐*actinin^+^ staining and formation of striated myofibrils (Fig. 1B, insert). Further depletive passaging successively enriched and promoted the expansion of a second presumptive progenitor population and loss of terminally differentiated cells from the culture. When seeded at low density, these cells expanded and reformed colonies, which when further passaged, resulted in largely homogenous monolayers. These cells could be passaged up to and beyond p24 (equivalent to 30 days), with no change in proliferative ability and growth kinetics, while maintaining homogenous expression of Nkx2‐5 and Tbx5, along with Irx4 and Gata4 that was not restricted to any specific subpopulation (Fig. 1C). Gene expression analysis (GEA) across the reprogramming time course confirmed the sustained upregulation of CP markers when compared with CF, further indicating the formation and expansion of progenitors (Fig. 1D).

Although an expandable progenitor population was obtained, it was unclear which subpopulation they originated from, be it from truly reprogrammed fibroblasts/myofibroblasts or simply expansion of pre‐existing CPs, which although scarce, may exist in coculture with fibroblast cultures. Given that PDGFRα is highly expressed in CFs, we performed lineage tracing using Pdgfra‐CreER‐ROSA26R^CAG‐LSL‐EGFP^ mouse line (Supporting Information Fig. S1E). Immunostaining of the heart sections confirmed PDGFRα expression exclusive of SM‐myosin heavy chain (SM‐MHC), indicating that PDGFRα can be used to mark CF populations, permitting their isolation without nascent SM contamination (Supporting Information Fig. S1F). Quantification of PDGFRα‐GFP^+^ cells by flow cytometry also revealed a near‐identical distribution of PDGFRα^+^ cells as the wild type that also do not express SM‐MHC (Supporting Information Fig. S1B vs. S1G). Treatment of PDGFRα‐GFP^+^ cells with SB431542 generated Nkx2‐5^+^ colonies, which are also GFP^+^, confirming that the likely origin of Nkx2‐5^+^ putative progenitors is a PDGFRα^+^/SM‐MHC^−^ CF population (Supporting Information Fig. S1H).

We next assessed the progenitor properties and differentiation potency of this second presumptive progenitor population in generating more mature cell types. GEA using a more comprehensive set of CP markers between these cells and the original fibroblasts revealed significant increases in the expression of key transcription factors and cell surface receptors with the exception of Flk1 and Mef2c (Fig. 1E). Analysis of cell surface markers by flow cytometry confirmed the increased expression of PDGFRα, CD105, CD44, CD29, and notably, Sca1 although increase in Flk1 protein expression was not detected (Fig. 1F). Consistent with the previous reports on CP marker expression, our cells did not express c‐Kit 17, 18. These results suggest that the progenitor cells we obtained are equivalent to MSCs or cardiac colony‐forming unit fibroblasts (cCFU‐F), 34. To further explore the differentiation potential of these progenitors, we induced differentiation by culturing the cells in low serum media supplemented with 5 ng/ml TGF‐β for 7 days. Morphological changes and positive staining for α‐SMA and calponin (Cnn1) revealed a propensity for the progenitors to undergo both myofibroblast and SM differentiation (Fig. 1G). Myofibroblasts possess features of both CF and SM, indicating that despite homogenous CP marker expression, there is heterogeneity in response to TGF‐β signaling within the population. Reassuringly, sorted Pdgfrα‐GFP^+^ fibroblasts that do not originally express SM‐MHC could give rise to SM‐MHC^+^ SM cells following reprogramming (Supporting Information Fig. S1I, S1J). Quantification of the number of colonies obtained from Alk5i reprogrammed cells further demonstrated a statistically significant increase compared with DMSO control (∼12 fold; Supporting Information Fig. S1K). Surprisingly, when the reprogramming was repeated with fibroblasts isolated from a ROSA26R^NKX2‐5‐Enhancer‐EGFP^ reporter mouse that carried a cardiac‐specific Nkx2‐5 enhancer, no GFP expression was detected, suggesting that the upregulation of Nkx2‐5 expression in this putative progenitor population is independent of CP‐specific enhancer activity (Supporting Information Fig. S1L). However, despite clear Nkx2‐5 upregulation, these cells were unable to convert into EC or CM, suggesting that this second progenitor population we generated is more restricted in its lineage commitment potential. This was confirmed in additional experiments using Alk5‐specific inhibitors RepSox or AZ12799734 35, 36 (Supporting Information Fig. S2A–S2E). Notably, ciSMPs retain expression of VIM, suggesting maintenance of mesenchymal properties, again indicative of an MSC/cCFU‐F identity albeit more lineage‐restricted (Supporting Information Fig. S2B). Thus, given the propensity for differentiation of these cells toward the SM lineage, either partially to myofibroblasts or directly to SM, we termed these cells as ciSMPs.

### CRISPR‐KO Screen Identifies Dmap1 as a Key Factor Regulating Reprogramming

**Figure 2:**
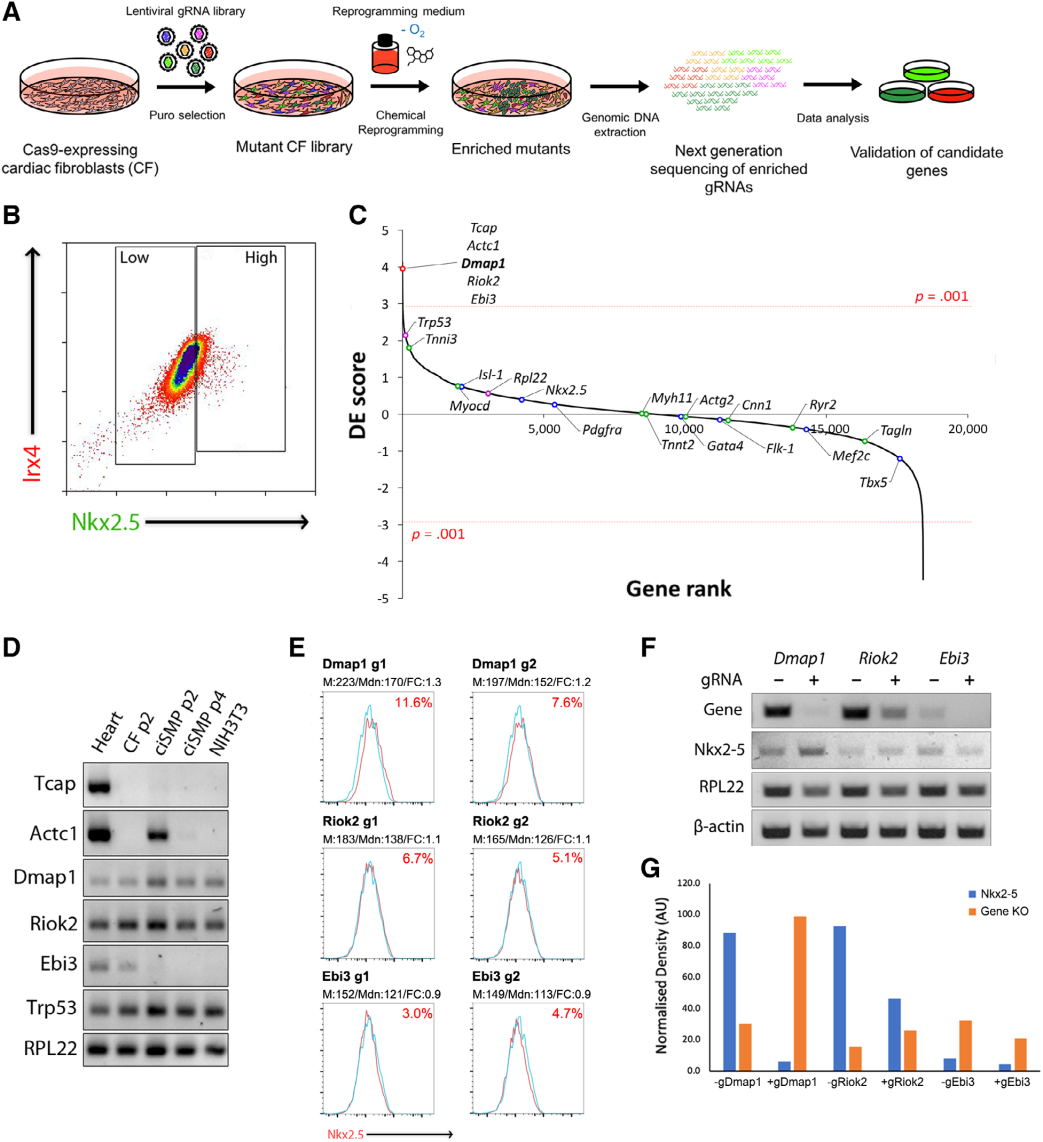
Clustered regularly interspaced short palindromic repeats (CRISPR)‐knockout screen for factors that enhance reprogramming. **(A):** Schematic outlining screening design and approach. **(B):** Fluorescence‐activated cell sorting (FACS) plot indicating markers used to distinguish nonreprogrammed (Nkx2.5^LOW^) and reprogrammed (Nkx2.5^HIGH^) cells with Irx4 as a costain for intact nuclei. Bulk population was gated as indicated, and genomic DNA was extracted from sorted populations for sequencing. **(C):** Ranking of genes based on depletion/enrichment (DE) score calculated from *p* values generated by model‐based analysis of genome‐wide CRISPR‐Cas9‐knockout. Top three hits are highlighted in red, CP markers are highlighted in blue, smooth muscle/cardiomyocyte marker highlighted in green, and housekeeping genes in purple. *p*‐value cut offs where *p* < .001 were used to distinguish statistically significant hits, where *n* = 3. **(D):** Semiquantitative gene analysis of expression of top hits and housekeeping controls in heart tissue, CF, cardiac progenitor, and NIH3T3 cells. **(E):** FACS staining for Nkx2.5 in ciSMP carrying single gRNA against top hits (red) verses nontargeting control (blue). M and Mdn represent mean and median values, respectively. FC represents fold change calculated using M and Mdn values from the empty control (M: 167, Mdn: 131). **(F):** Semiquantitative gene expression analysis in ciSMP carrying single gRNA against Dmap1, Riok2, and Ebi3. “Gene” indicates expression of gene targeted by respective gRNA. **(G):** Corresponding densitometry analysis of data in **(F)** normalized to RPL22. See also Supporting Information Figure S3. Abbreviation: CF, cardiac fibroblast; ciSMP, chemically induced smooth muscle progenitor; Dmap1, DNA methyltransferase 1‐associated protein 1; gRNA, guide RNA.

CRISPR‐Cas9 knockout screens are well suited to deciphering the molecular intricacies behind the phenotypic changes associated with reprogramming. Therefore, we optimized and scaled up our reprogramming protocol to conduct a genome‐wide screen to identify factors or pathways that regulate this process (Fig. 2A). Following isolation of Cas9‐CF from Cas9 mice, we first confirmed that Cas9 activity was preserved by transducing the cells with a lentiviral construct, which encoded for BFP‐2A‐GFP concurrent with self‐targeting GFP‐gRNA. Robust Cas9 activity was demonstrated by a complete shift in cell population from BFP^+^/GFP^+^ to BFP^+^ alone when compared with control (Supporting Information Fig. S3A). Hence, delivery of the fully optimized gRNA library followed by selection permitted the generation of a mutant CF library, which was then subjected to reprogramming. Given that Nkx2‐5 upregulation was the most clear and consistent hallmark indicating reprogramming, we defined our phenotypic parameter in sorting reprogrammed (Nkx2‐5^HIGH^) and non‐reprogrammed (Nkx2‐5^LOW^) cells via intracellular staining followed by flow cytometry analysis (Fig. 2B). Additionally, we verified that our antibody was capable of detecting the protein in formaldehyde‐fixed NIH3T3 cells overexpressing Nkx2‐5 (Supporting Information Fig. S3B). Cells were also stained with Irx4, which is expressed at high levels in both fibroblast and progenitor populations and was used as a costain to mark intact nuclei. Sorted cells were then subjected genomic DNA extraction and then gRNA read‐counts were obtained via next‐generation sequencing. The data were subsequently processed via MAGeCK, a computational tool that statistically evaluates whether certain guides are significantly enriched or depleted between two populations. Combining the enrichment and depletion *p* values for each gene allowed the computation of the depletion‐enrichment (DE) score (Supporting Information Fig. S3C). A positive DE score indicates that loss of that particular gene is advantageous for reprogramming, whereas conversely a negative score identifies genes that are essential for the process (Fig. 2C; Supporting Information Fig. S3C). In assuming that Nkx2‐5 expression accurately reflects reprogramming efficacy, guides against known CP, SM, and CM markers were neither significantly enriched nor depleted, implying that although enhanced and sustained CP marker expression is a hallmark of progenitor derivation, loss of their expression does not hinder nor influence progenitor formation. Loss of the classic tumor suppressor gene *Trp53* enhances cell proliferation in CF leading to enrichment of *Trp53*‐targeting gRNA, yet had no significant impact on Nkx2‐5 expression, and hence reprogramming. Equally, suppression of markers and structural proteins attributed to further differentiated stages does not affect the reprogramming efficiency as indicated by Nkx2‐5 expression levels. To assess whether the top five hits were truly relevant to the observed phenotype, we performed semiquantitative GEA to gauge their expression in the heart and across the reprogramming process (Fig. 2D). *Tcap* and *Actc1* expression was primarily restricted to the heart and, by inference, CM alone, suggesting that as structural proteins, they do not play a significant role in the conversion process. Detection of *Actc1* expression during early enrichment was expected given that the colonies can spontaneously give rise to both EC and CM upon prolonged reprogramming (Fig. 1B; Supporting Information Fig. S1C). However, *Dmap1* and *Riok2* were found to have sustained expression before and after the reprogramming process, suggesting that loss of function may have an effect in enhancing Nkx2‐5 expression. To further validate the data from our screen, we performed experiments using two individual gRNAs for each of the top three candidates with the highest positive DE score that did not encode for structural proteins. Genes that when lost lead to an increase in Nkx2‐5^+^ cells of greater than 5% when compared with the empty control was used to demonstrate improved reprogramming efficiency. Out of the top three hits, guides which target *Dmap1* (g2 and g3) consistently enhanced Nkx2‐5 expression by 7.6% and 11% compared with a baseline elevated expression of 5% during routine reprogramming (Fig. 2E). Assessing the effectiveness of the gRNAs used against *Dmap1*, *Riok2*, and *Ebi3* by semiquantitative PCR further confirmed both the effectiveness of Dmap1 gRNA‐mediated gene knockout and effects upon Nkx2‐5 expression (Fig. 2F, 2G), prompting us to further investigate the contribution of Dmap1 to progenitor properties.

### Loss of Dmap1 Enhances the Expression of CP Genes in ciSMP

**Figure 3:**
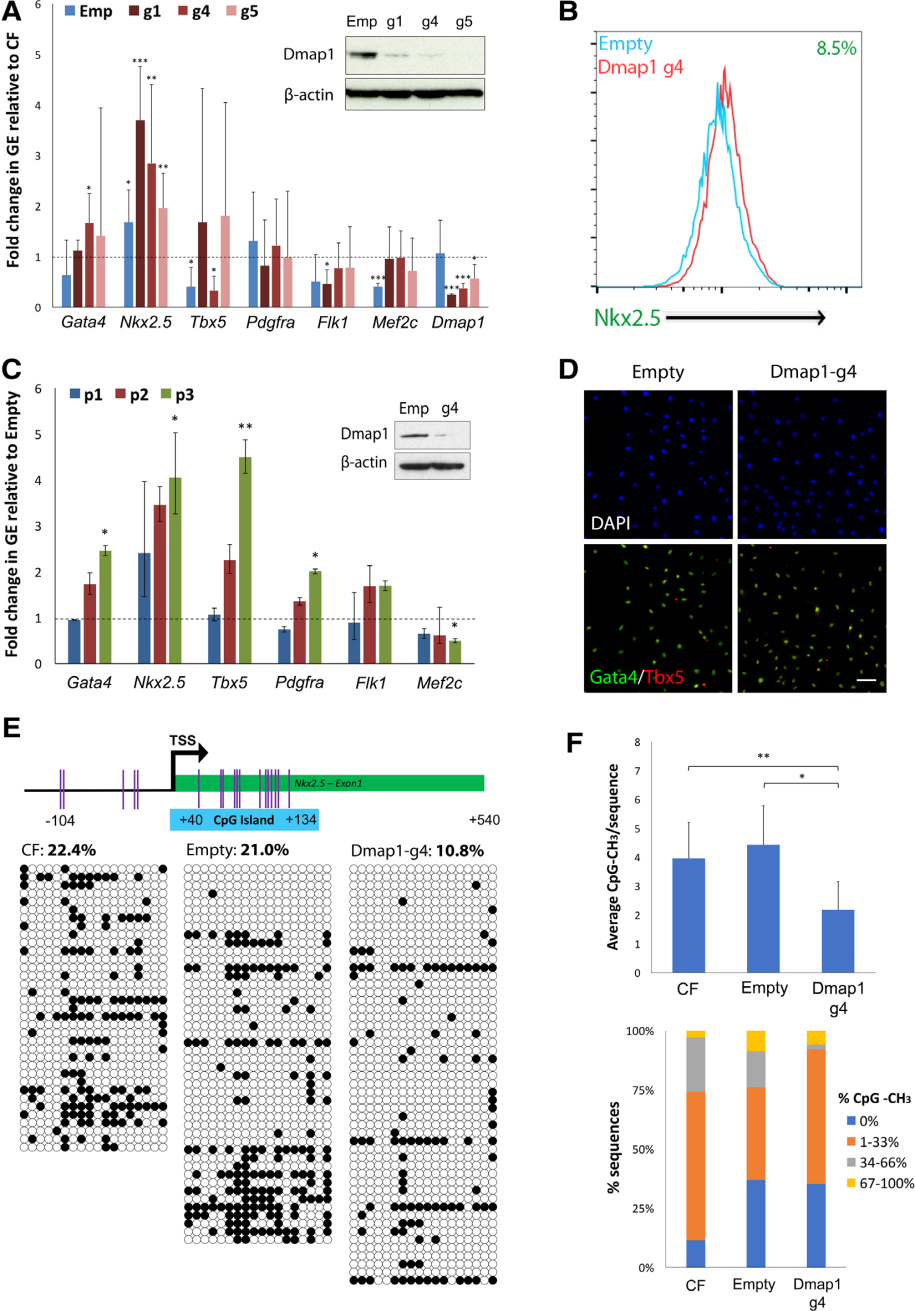
Characterization of Dmap1‐knockout phenotype in chemically induced smooth muscle progenitors (ciSMPs). **(A):** Immunoblot of ciSMP carrying three different guides against Dmap1 relative to empty control and average gene expression of CP genes in these ciSMP over the first five passages relative to CF. Dotted line indicates relative fold change in expression in CF, where RGE = 1. Error bars indicate SD. **(B):** Fluorescence‐activated cell sorting staining for Nkx2.5 in ciSMP carrying g4‐guide RNA (red) versus empty control (blue). Percentage shift in Nkx2.5 expression as indicated. **(C):** Gene expression analysis of cardiac progenitor markers in Dmap1‐g4 cells relative to empty control across the first three passages. Immunoblot confirms sustained loss of Dmap1 expression in Dmap1‐g4 ciSMP. **(D):** Immunostain of cells in **(B)** depicting costaining of Gata4 and Tbx5. Scale bar indicates 100 μm. **(E):** Methylation analysis of the *Nkx2.5* promoter region in CF, empty, and Dmap1‐g4 ciSMP. **(F):** Summary of data in **(E)** depicting average number of methylated CpG/sequence and frequency of total methylation across all sequences. * and ** indicate *p* < .05 and *p* < .01, respectively, as calculated by Student's *t* test, where *n* = 3. See also Supporting Information Figure S4. Abbreviations: CF, cardiac fibroblast; DAPI, 4′,6‐diamidino‐2‐phenylindole; Dmap1, DNA methyltransferase 1‐associated protein 1; GE, gene expression; TSS, transcription start site.

We elected to analyze the effects of Dmap1 loss in ciSMP given that we could not maintain the potency of the initial progenitors under reprogramming conditions. To rule out the possibility of off‐target effects, we first repeated the reprogramming process using three additional guides (g1, g4, and g5) against *Dmap1*. Dmap1 loss was confirmed both on the mRNA and protein level as indicated for all guides (Fig. 3A, 3C). GEA of CP markers within the first five passages following reprogramming revealed an overall increase in the expression of specific marker genes, most significantly in Nkx2‐5 relative to original CF although other markers appeared more variable (Fig. 3A). Given that multiple gRNAs against *Dmap1* were able to reproduce the same phenotype, we concluded that the observed Nkx2‐5 upregulation was a genuine consequence of *Dmap1* loss and focused on the use of ciSMP derived from CF that were transduced with g4 for subsequent analyses (henceforth g4‐ciSMP vs. empty). CRISPR‐Cas9 editing of the *Dmap1* locus via g4‐gRNA was confirmed via tracking of indels by decompositions sequencing (Supporting Information Fig. S4A) and enhanced Nkx2‐5 expression was again confirmed via intracellular flow cytometry, with 8.5% of the cells being Nkx2‐5^HIGH^ (Fig. 3B). A more robust assessment of CP gene expression across the first three passages in g4‐ciSMP revealed a significant increase in all markers again with the exception of Flk1 and Mef2c when compared with empty control (Fig. 3C). Furthermore, these markers are uniformly coexpressed in both control and knockout ciSMP, indicating that the difference observed is not because of the expansion of a subpopulation that highly express Gata4 or Tbx5 (Fig. 3D).

In identifying Nkx2‐5 upregulation as the defining and most consistent effect of *Dmap1* loss, we next explored the mechanism by which this is achieved. Dmap1 is known to associate with and regulate the activity of DNA methyltransferase‐1, which in turn regulates gene expression through promoter methylation. In assessing the methylation status of the Nkx2‐5 promoter in CF, empty and g4‐ciSMP, we observed that loss of Dmap1 significantly decreased Nkx2‐5 promoter methylation (Fig. 3E, 3F). The overall methylation reduced by 50% when compared with empty control and decreased from an average of 4 to 2 CpG methylated residues per sequence. Notably, this decrease appears to be driven by an overall reduction of moderately methylated sequences (33%–66% to 1%–33%) rather than a complete loss in methylation across all CpG residues (Fig. 3F). Loss of *Dmap1* does not reduce the methylation of the deleted in azoospermia‐like 1, a prenatal, germ‐cell‐specific gene whose expression is strongly repressed by promoter methylation in somatic cells (Supporting Information Fig. S4B), suggesting locus‐specific promoter methylation changes. Overall, these data suggest that modulation of Nkx2‐5 via promoter methylation is a key mechanism by which ciSMP properties are controlled.

### Loss of Dmap1 Attenuates Myofibroblast and SM Differentiation

**Figure 4:**
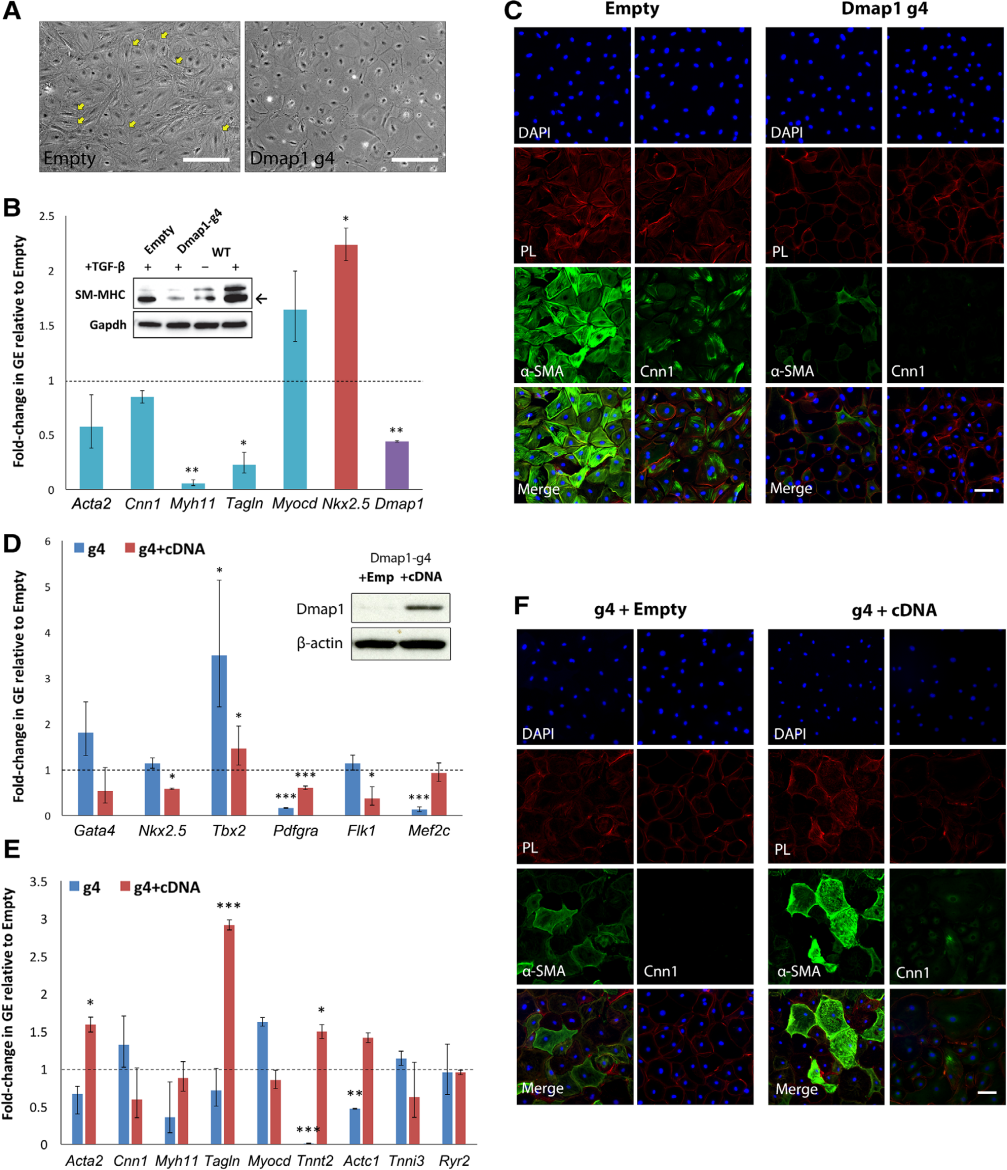
Effect of Dmap1‐knockout (KO) on the further differentiation of chemically induced smooth muscle progenitor (ciSMP). **(A):** Phase contrast of control and Dmap1‐KO ciSMP differentiated to smooth muscle (SM). Arrows indicate cells that display formation of contractile filaments that are absent in g4‐derived SM. **(B):** Gene expression analysis (GEA) of SM markers (blue) in KO‐derived SM relative to empty control (dotted line). Error bars indicate SD. Immunoblot confirming induction of SM‐MHC in WT cells upon TGF‐β treatment and loss of SM‐MHC expression in absence of Dmap1. **(C):** Immunostain of SM derived from empty and KO‐ciSMP for α‐SMA and Cnn1. **(D):** GEA of CP markers KO‐ciSMP (g4) and g4‐ciSMP transduced with Cas9‐resistant Dmap1‐cDNA (g4 + cDNA) relative to respective empty controls. Immunoblot confirming restoration of Dmap1 expression. **(E):** GEA of SM and cardiomyocyte markers in g4 and g4 + cDNA cells relative to respective empty controls. **(F):** Immunostain of SM derived from ciSMP outlined in **(D)** for α‐SMA and Cnn1. Scale bars indicate 100 μm. *,**, and *** represent *p* < .05, *p* < .01, and *p* < .001, respectively, when compared with relative empty control, where *n* = 3. Abbreviations: α‐SMA, alpha‐smooth muscle actin; Cnn1, calponincalponin; DAPI, 4′,6‐diamidino‐2‐phenylindole; Dmap1, DNA methyltransferase 1‐associated protein 1; GE, gene expression; PL, phalloidin; SM‐MHC, smooth muscle‐myosin heavy chain; TGF‐β, transforming growth factor‐β; WT, wild type.

We next explored whether *Dmap1* loss impacts ciSMP differentiation. Although cells expressing control gRNA underwent consistent morphological changes upon induction, *g4‐*ciSMP failed to form the characteristic actin filaments associated with myofibroblast and SM induction (Fig. 4A). Correspondingly, GEA for SM markers revealed a significant decrease in MHC 11 (Myh11) and transgelin (Tagln) expression when compared with SM derived from empty ciSMP, which was further confirmed by SM‐MHC immunoblot (Fig. 4B). Although not significantly reduced on the mRNA level, there was clear reduction in both α‐SMA and Cnn1 expression at the protein level as evidenced by immunostaining (Fig. 4C). To demonstrate that this defect is because of the absence of Dmap1, we introduced cDNA encoding Cas9‐resistant Dmap1 into g4‐ciSMP and analyzed whether the defect in SM differentiation could be rescued (Fig. 4D). GEA of ciSMP transduced with Dmap1 cDNA revealed a significant suppression of CP markers, notably of Nkx2.5, although differentiated cells derived from both g4 and g4 + cDNA ciSMP revealed a partial restoration of differentiation efficacy, particularly in *Acta2* and *Tagln* expression (Fig. 4E). Immunostaining of g4 + cDNA‐derived SM demonstrated restoration of α‐SMA and Cnn1 expression, albeit to the lesser extent than empty control (Fig. 4C, 4F).

### Dmap1 Promotes Retention of SM Potency via Suppression of E‐Cadherin

**Figure 5:**
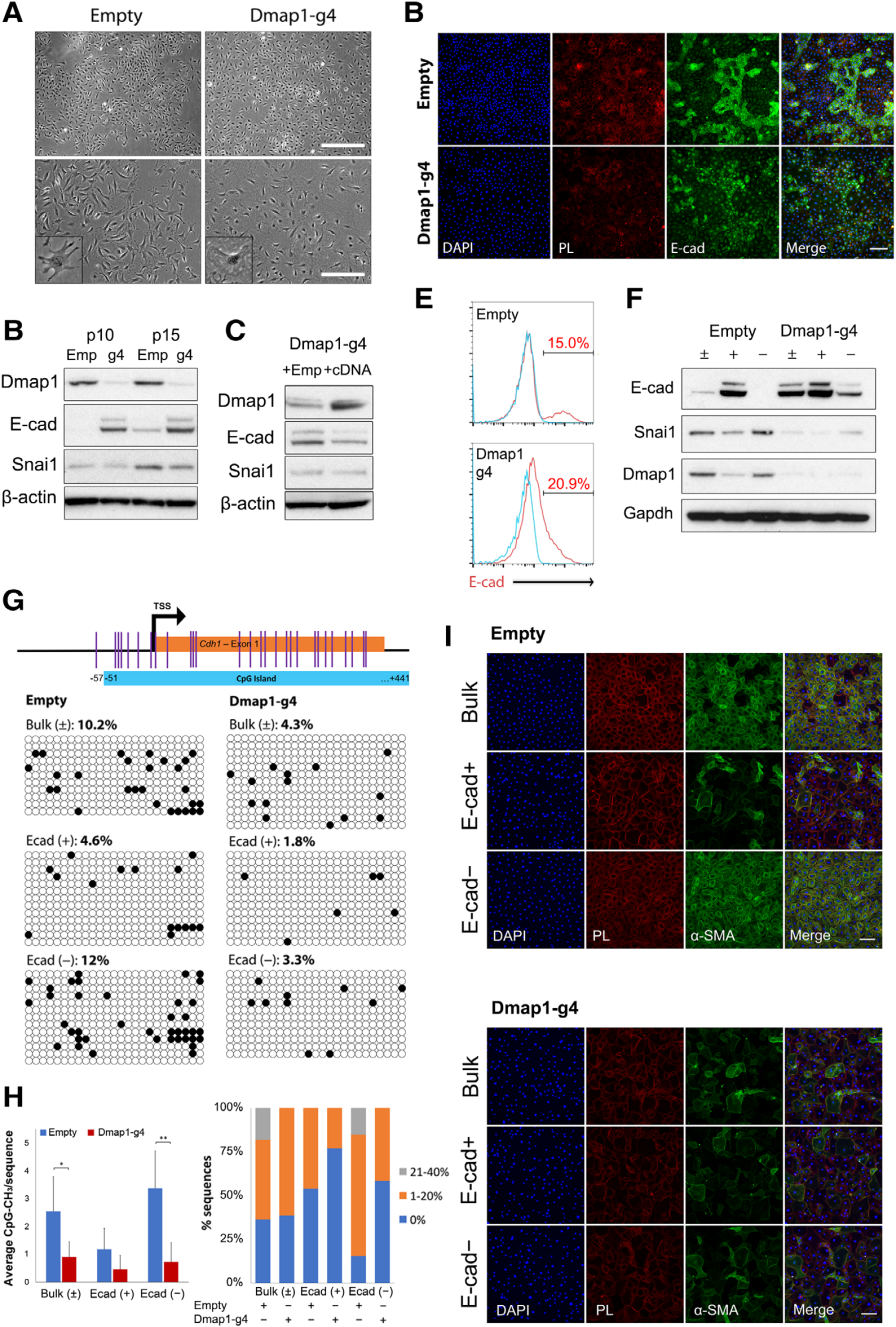
Dmap1 attenuates smooth muscle (SM) induction via suppression of EMT. **(A):** Phase contrast of control and Dmap1‐knockout (KO) chemically induced smooth muscle progenitor (ciSMP). Inserts detail single cells demonstrating contrasting morphologies. **(B):** Immunoblot assessing the expression of Dmap1 and EMT modulators E‐cad and Snai1. **(C):** Immunoblot of rescue experiment and impact on EMT‐related protein expression **(D):** Immunostain of ciSMP derived from empty and g4‐cardiac fibroblast for E‐cad. **(E):** Flow cytometry of cells stained for E‐cad in **(D)**. **(F):** Immunoblot of cells sorted from **(E)** for Dmap1 and EMT‐related proteins. ±, bulk; –, E‐cad^−^ or E‐cad^LOW^; and +, E‐cad^+^ or E‐cad^HIGH^ populations in empty and Dmap1‐KO. **(G):** Methylation analysis of E‐cad promoter region from cells sorted in **(E)**. **(H):** Summary of data in **(G)** depicting average number of methylated CpG/sequence and frequency of total methylation across all sequences. * and ** indicate *p* < .05 and *p* < .01, respectively, as calculated by Student's *t* test. **(I):** Immunostain of cells sorted from **(E)** that have been subjected to SM differentiation and stained for α‐SMA. Scale bars indicate 100 μm. See also Supporting Information Figure S5. Abbreviations: α‐SMA, alpha‐smooth muscle actin; DAPI, 4′,6‐diamidino‐2‐phenylindole; Dmap1, DNA methyltransferase 1‐associated protein 1; E‐cad, E‐cadherin; PL, phalloidin; TSS, transcription start site.

During routine propagation of both control and g4‐ciSMP, we observed that there were noticeable differences in terms of their cell morphology, with control cells generally adopting a more mesenchymal appearance, opposed to the more epithelial features observed in the knockout cells (Fig. 5A). Given that proper regulation of epithelial‐mesenchymal transition (EMT) is required for proepicardial cells to form SM 37, 38, we analyzed E‐cadherin (E‐cad) and Snai1 expression and found significant upregulation of E‐cad in Dmap1‐KO cells, which was maintained across passages (Fig. 5B). Restoration of *Dmap1* expression in the knockout line reduced E‐cad expression, clearly demonstrating the suppression of E‐cad in the presence of Dmap1, although this is independent of Snai1 activity which remained unchanged (Fig. 5C). Equally, further analysis of the signaling dynamics downstream of TGF‐β signaling strongly correlates decreased expression of E‐cad with TGF‐β activity, particularly evident in control cells (Supporting Information Fig. S5A, S5B). Indeed, although Dmap1‐KO g4 cells are still responsive to TGF‐β signaling, this effect is nonetheless blunted, sustaining high levels of E‐cad expression despite the presence of active TGF‐β signaling (Supporting Information Fig. S5B, S5C). Immunostaining and flow cytometry revealed that specific E‐cad^+^ populations did exist in the largely E‐cad^−^ control cells, whereas Dmap1‐KO cells were all E‐cad^+^ and demonstrated a broad range of expression (Fig. 5D, 5E). To further investigate the properties of the E‐cad^+^ subpopulation, we sorted E‐cad^+^ cells and compared the expression of E‐cad, Snai1, and Dmap1 (Fig. 5F). The E‐cad^+^ fraction from control cells showed decreased expression not only of Snai1 but also Dmap1, suggesting an implicit role for Dmap1 in repressing E‐cad expression. However, cells appear to revert toward bulk distribution levels in terms of E‐cad expression following passaging, suggesting the presence of dynamic processes that regulate Dmap1 expression (Supporting Information Fig. S5D). Notably, in the control population, 43% of the original E‐cad^+^ cells had lost expression of E‐cad, although only 2.5% of the original E‐cad^−^ population became E‐cad^+^, suggesting an inclination toward an E‐cad^−^ state when Dmap1 is present. Methylation analysis of the *Cdh1* promoter region showed the overall decrease in methylation in g4‐ciSMPs as well as a decrease in E‐cad^+^ cells sorted from empty ciSMP, confirming the correlation of promoter methylation with E‐cad expression (Fig. 5G vs. 5F). Sorted cells were subjected to SM differentiation, which further confirmed the role Dmap1 plays in suppressing mesenchymal properties (Fig. 5I). Control E‐cad^−^ cells were able to fully differentiate into SM, whereas E‐cad^+^ progenitors isolated from the same bulk population failed to differentiate, akin to Dmap1‐KO progenitors. Full suppression of *Cdh1* expression by Dmap1 appears to be required for commitment to the SM fate, as even E‐cad^−^ Dmap1‐KO cells that have a relatively low level of E‐cad expression could not form SM. Together, these data suggest a critical role for Dmap1 not only in the preservation of ciSMP properties via Nkx2‐5 but also in regulating the latter commitment of progenitors into SM.

## Discussion

### Chemical Reprogramming Induction of Progenitors

The defining properties of progenitors that ensure self‐renewal while maintaining a differentiation permissive state also define their prospects for therapeutic use. However, in terms of cardiac regeneration, this approach has been hampered by a lack of consensus as to what constitutes a true CP and whether such progenitors can be generated without transgene‐dependent induction of the cardiac transcriptional program. Both direct and indirect chemical reprogramming approaches have clearly demonstrated that it is entirely possible to induce reprogramming in the absence of transgenes, and as we have shown, it is also capable of generating progenitor populations. Early on in the reprogramming process, these cells display potency in generating EC and CM, although the functionality of these cells requires further investigation. However, sustained culture under reprogramming conditions compromise the potency of these cells as certain markers such as Flk1 and Mef2c become downregulated, thus explaining the inability of this second population of progenitors to generate CM or EC despite retaining the capacity for SM differentiation. The spontaneous nature of differentiation late in the reprogramming process suggests that culture conditions will need to be further optimized in order to preserve both progenitor potency and self‐renewal.

### ciSMP Identity in Relation to MSC and cCFU‐F

We have found that ciSMP share many similar features to cCFU‐F, such as maintenance of VIM, CD44 expression, and mesenchymal morphology, albeit with a more restricted repertoire for further differentiation. cCFU‐Fs are of proepicardial origin and demonstrate a broad spectrum of germ layer potency, including those beyond the cardiac lineage 34. Although our protocol may have the potential of facilitating direct expansion of pre‐existing cCFU‐F in the primary culture, this appears highly unlikely, given that cCFU‐Fs are exceptionally rare (<0.1% of Sca1^+^/PDGFRα^+^ population) and more importantly, that TGF‐β signaling rather promotes cCFU‐F colony formation and self‐renewal while maintaining lineage potency 39. Our reprogramming conditions are heavily dependent upon TGF‐β suppression. Thus, given the different responses to TGF‐β signaling and capacity for differentiation, cCFU‐F and ciSMP cannot be regarded as equivalent, despite sharing key molecular features. Although ciSMPs are more lineage‐restricted, they retain some degree of plasticity, given that cells sorted for either positive or negative E‐cad expression demonstrate a tendency for reversion back to bulk population distribution after passaging. It remains unclear how *Dmap1* expression is dynamically regulated; however, there is a clear inverse correlation between Dmap1 and E‐cad expression, further suggesting that promoter methylation is also a dynamic process, at least with regard to *Cdh1*.

### Promoter Methylation and Regulation of Gene Expression

We have consistently observed that even seemingly subtle changes in promoter methylation appear to have significant effects on gene expression and hence resultant phenotype. In the case of Dmap1, a 50% reduction in this methylation resulted in a two‐ to fourfold increase in the expression of Nkx2‐5. Equally, a 75% reduction in *Cdh1* methylation was observed in the E‐cad^−^ population between empty and Dmap1 knockout cells, strongly implicating Dmap1 activity in the regulation of Cdh1 promoter methylation. In both cases, this results in a strong phenotype, namely the inability to differentiate toward the SM lineage in the absence of Dmap1 and vice versa in the case of E‐cad^−^ ciSMP and Cdh1 expression. Similarly, subtle changes linked to strong phenotypes have also been observed in transcription‐factor‐based approaches, whereby CM‐specific genes such as *Myh6* and *Nppa* remain at least 50% methylated even after 3 days of reprogramming to CM 40. Comparison between empty and transduced cells also revealed that despite an overall promoter methylation change of only 5% to 10%, specific CpG islands demonstrate up to 40% demethylation when compared with control, suggesting that changes at specific islands can have a disproportionate and perhaps cumulative influence on the gene expression 40. Furthermore, it has been demonstrated that there is a strong correlation among CpG island methylation, distance from the TSS, and gene expression, and therefore, analysis of these regions is most likely to reveal the methylation associated changes in the gene of interest 41. We have observed that this is indeed the case for Nkx2‐5 and Cdh1, which correspondingly have a clearly defined CpG island flanking the TSS. It is however important to note that promoter methylation is only one of many factors influencing gene expression during reprogramming and that complete resolution of epigenetic suppression mechanisms is required to fully unlock progenitor potency.

### TGF‐β Signaling and Reprogramming

The basis of the chemical reprogramming approach is that small molecules can act to alter the signaling and epigenetic environment that facilitates the upregulation of lineage‐specific genes that establish a new cell identity. TGF‐β signaling has been extensively studied both in the cancer and developmental context, playing key roles in the control of tumor metastasis, cell differentiation, and epithelial‐mesenchymal interconversion 42. Suppression of this pathway sustains the mesenchymal properties of both fibroblasts and ciSMP as reflected by retention of fibroblast properties (CD44^+^, VIM^+^) concurrent with increased Nkx2‐5 expression. However, the effectiveness of this process is dependent on the activity of Dmap1, as Dmap1 acts to promote this process via the downregulation of E‐cad expression. Concurrently, absence of Dmap1 antagonizes ciSMP mesenchymal properties because of the aberrant E‐cad expression, detrimentally inhibiting further differentiation into SM. Although the *Cdh1* promoter is not heavily methylated under ciSMP culture conditions, Dmap1 is nonetheless required for full and sustained suppression of E‐cad before SM induction. Consequently, although Dmap1‐KO cells do demonstrate features that imply improved progenitor properties akin to embryonic equivalents (i.e., increased Nkx2‐5), this might actually represent ciSMPs that are trapped in a nondifferentiation permissive, yet self‐renewing state. Indeed, a similar population of ciSMP can be found in cells where Dmap1 expression is low, indicating the necessity of Dmap1‐mediated suppression of E‐cad before SM induction. This reflects the limitations of our screening approach, as although factors can be uncovered that enhance progenitor properties in terms of CP marker expression, the same factors could be required for access to further differentiation and is indistinguishable by a single selection criterion. Additionally, it is well established that induction of TGF‐β enhances myofibroblast differentiation and deposition of extracellular matrix that ultimately leads to fibrotic scar deposition in vivo 43. Therefore, suppression of TGF‐β signaling may act to preferentially stimulate fibroblast transdifferentiation into progenitor cells that can then terminally differentiate to restore the normal tissue architecture. In most cell and tissue culture studies, atmospheric oxygen concentration (21%) has been used. Although this is usually termed “normoxic,” it is in actuality hyperoxic as the oxygen concentration is much lower in situ in most tissues. For example, in the well‐perfused normal heart, the oxygen concentration ranges from 4% to 14% and is lower in the ischemic and border areas of the diseased heart 44, 45. We observe that normoxic conditions are detrimental to the reprogramming process, suggesting that hypoxic conditions that mimic physiological levels may also act to stimulate and stabilize the formation of CP when TGF‐β signaling is suppressed. This certainly appears to be the case for ciSMP expansion, which additionally possess many markers of MSC that are known to demonstrate improved proliferation and self‐renewal properties under hypoxia.

### Dmap1 and Preservation of Mesenchymal Potency

Dmap1 was originally identified as a cofactor that regulates Dnmt‐1‐mediated DNA methylation and has been documented to have distinctive roles in reshaping the epigenetic environment during development 46, whereas Dnmt‐1 itself has been found to be essential in preserving progenitor function, notably in somatic tissues 47. Correspondingly, we have shown that Dmap1 acts directly to repress E‐cad expression, which is fundamental in preserving mesenchymal potency and access to latter SM fates. This is independent of classical EMT suppression via Snai1/2, as altering Dmap1 expression does not induce significant changes in Snai1 expression. However, there is clear correlation in that low levels or loss of Dmap1 expression is typically concurrent with reduced Snai1 expression. This suggests that Dmap1 is a key component in the regulative pathways that suppress epithelial properties in conjunction with Snai1. In addition to Dnmt‐1 regulation, Dmap1 is also an integral part of the NuA4 histone acetyltransferase complex 48, 49 and may predictably have other repressive functions beyond promoter methylation, although in this context, suppression of E‐cad alone is sufficient in preventing loss of mesenchymal properties. Indeed, we have shown that specific and subtle changes to promoter methylation can have a significant impact on progenitor state maintenance and differentiation. Given the importance of Dmap1 in inhibiting aberrant gene expression and tumorigenesis, induction of progenitor reprogramming via chemically induced epigenetic modification warrants further investigation.

## Summary

This study validates the use of a chemical reprogramming approach in generating CP populations of varying potency. Although initial progenitor populations are capable of differentiation toward CM and EC lineages, this is rapidly lost upon extended culture under reprogramming conditions, resulting in an expandable progenitor population that can only give rise to myofibroblast and SM lineages, termed ciSMP. Utilizing CRISPR‐KO screening, we uncovered a role for Dmap1 in mediating both progenitor induction and differentiation of ciSMP via regulation of promoter methylation of Nkx2‐5 and Cdh1, respectively. In order to realize the use of this approach in therapeutic reprogramming, further studies will be required to uncover the correct conditions needed to retain maximal progenitor potency while retaining self‐renewal. We have demonstrated that promoter methylation is critical for this process, and predictively, envision that it plays a broader role in sustaining progenitor potency.

## Author Contributions

J.S.L.Y.: conception/design, collection and assembly of data, data analysis and interpretation, manuscript writing, final approval of manuscript; G.P.: conception/design, collection of data, data analysis and interpretation; C.L.: conception/design, data analysis and interpretation; A.M.: collection of data, data analysis and interpretation; L.D.: provision of study material or patients, data analysis and interpretation; A.T.P.: administrative support, provision of study material or patients; M.B.‐Y., B.S.R.: administrative support, data analysis and interpretation; E.M.H.: conception/design, assembly of data, financial support, data analysis and interpretation, final approval of manuscript; Q.‐D.W., K.Y.: conception/design, financial support, data analysis and interpretation, manuscript writing, final approval of manuscript.

## Disclosure of Potential Conflicts of Interest

C.L., L.D., A.T.P., M.B.‐Y., B.S.R., Q.‐D.W. are former or current employees of AstraZeneca. A.T.P. is now currently employed at Sanofi. E.M.H. declared research funding from Karolinska Institutet/AstraZeneca Integrated CardioMetabolic Centre. The other authors indicated no potential conflicts of interest.

## Supporting information


**Figure S1:** Verification of cardiac fibroblast origin and contribution to reprogramming.
**A.** Immunostaining in CF for vimentin (VIM) and α‐SMA. **B.** FACS staining of CFs with Thy1.2 and PDGFRα. **C.** Phase contrast of CF cultures at d13 of reprogramming in the presence of DMSO or SB431542. Scale bars indicate 100 μm. **D.** Immunostain of differentiated colony at d21 post‐reprogramming for endothelial and cardiomyocyte markers. **E.** Schematic detailing lineage tracing experiments utilizing Pdgfra‐CreER‐ROSA26R^CAG‐LSL‐EGFP^ mouse strain and Nkx2‐5 enhancer activity assay using ROSA26R^NKX2‐5‐ENHANCER‐BP‐EGFP^ strain. **F.** Immunostain of heart sections prepared from mouse in (**E**, lineage tracing). **G.** Flow cytometry analysis of CF isolated from mouse in (**E**) for PDGFRα expression. **H.** Immunostain of sorted CF reprogrammed in the presence of DMSO or Alk5 inhibitor for Nkx2‐5. **I.** Immunostain of sorted CF from (**E**, lineage tracing) for SM‐MHC. **J.** Immunostain of progenitors derived from sorted CF cultured under SM differentiation conditions for SM‐MHC. Scale bars indicate 50 μm. **K.** Number of colonies per well derived from sorted CF reprogrammed in DMSO or Alk5i and percentage of SM‐MHC positive cells obtained from either DMSO or Alk5i derived cells. **L.** Flow cytometry analysis for EGFP expression in cells that have been isolated from mice (E, enhancer activity) and subjected to reprogramming under DMSO or Alk5 inhibitor. Red indicates non‐fluorescent control, green indicates sample.
**Figure S2:** Validation of Alk5 inhibition‐induced reprogramming protocol.
**A.** Phase contrast images depicting reprogramming process conducted with alternative Alk5 inhibitors AZ12799734 and RepSox. **B.** ciSMP arising from reprogramming detailed in (**A**) stained for Nkx2‐5 and VIM. **C.** Further immunostaining for Isl‐1 and Gata4 in enriched ciSMP arising from (**A**). **D.** SM differentiation in the absence of TGF‐β demonstrating low differentiation efficiency. **E.** SM differentiation of ciSMP isolated via (**A**) and stained for smooth muscle myosin heavy chain (SM‐MHC). Scale bars indicates 50 μm.
**Figure S3:** Optimization of CRISPR‐KO screen.
**A.** Assessment of Cas9 activity in CF isolated from Cas9 mice. Cells were transduced with construct containing BFP‐2A‐GFP with empty guide or self‐targeting guide against GFP. Presence and measure of Cas9 activity is indicated by the shift in double positive population to single BFP+ 3 days post transduction. **B.** Testing of antibody specificity in paraformaldehyde‐fixed NIH3T3 cells overexpressing Nkx2‐5 for use in subsequent FACS. 1° indicates primary antibody. Scale bars indicates 100 μm. **C.** MAGeCK output from gRNA read‐counts obtained from next generation sequencing computing abundance of read‐counts for a particular gene in Nkx2‐5^LOW^ vs Nkx2‐5^HIGH^ populations. Id: Gene, num: no. of gRNA targeting gene in library, p.neg/pos: associated P‐value for depletion/enrichment, fdr.neg/pos: false discovery rate of hit as depleted/enriched, rank.neg/pos: ranking by MAGeCK based on FDR, goodgrna.neg/pos: no. of gRNA against target gene that based on read counts can be characterized as working, DE score: score calculated as the summation of log10 p.neg and p.pos values where a positive value represents enrichment whilst negative represents depletion relative to Nkx2‐5^LOW^ population. DE score was used to rank the genes to produce the hit list and Figure 2C.
**Figure S4:** In‐depth characterization of Dmap1‐KO ciSMP.
**A.** Tracking of Indels by Decomposition (TIDE) analysis of g4‐gRNA mediated editing of the *Dmap1* locus versus unedited empty control. Boxes on respective chromatograms indicate PAM sequence, shaded regions indicate gRNA binding site. Arrow indicates single‐base adenine frameshift insertion, the most commonly identified modification in Dmap1‐g4 ciSMP (62.7%). **B.** Methylation analysis of the *Dazl* promoter in CF, empty and Dmap1‐g4 ciSMPs.
**Figure S5:** Response of ciSMP to TGF‐β signaling during SM differentiation.
**A.** Immunoblot of ciSMP differentiated overnight with TGF‐β or SB for E‐cadherin, Dmap1, Gapdh and Akt. Note that transfer of ciSMP into SM inductive conditions significantly alters the expression of Gapdh, however total Akt is unaffected. **B.** Densitometry measurements of (**A**) normalized to total Akt levels. **C.** Acute treatment of g4‐ciSMP with TGF‐β or SB. RM indicates cells that have been passaged into reprogramming media and left to plate down overnight, whilst “0” time point indicates cells passaged into SM differentiation media without TGF‐β or SB. “1” and “3” indicate 1 and 3 hours of treatment respectively. **D.** Flow cytometry analysis of E‐cadherin expression in control and Dmap1‐KO cells that have been passaged twice post‐sort.
**Table S2:** gRNA used to generate KO lines.
**Table S3:** Antibodies and dilutions used in this study.
**Table S4:** qRT‐PCR primers used in this study.
**Table S5:** Bisulfite cloning primers used in this study.

**Table S1:** Raw read counts and full screen gene list ranked by DE score.

## Data Availability

The data that support the findings of this study are available from the corresponding author upon reasonable request.
