# Supplementary material for: CRISPR‐Knockout Screen Identifies Dmap1 as a Regulator of Chemically Induced Reprogramming and Differentiation of Cardiac Progenitors
Source: Stem Cells. 2019 Apr 23;37(7):958–72. doi: 10.1002/stem.3012 (PMC6767549; doi:10.1002/stem.3012)
Supplement: Supplementary file 1 — Figure S1: Verification of cardiac fibroblast origin and contribution to reprogramming. A. Immunostaining in CF for vimentin (VIM) and α‐SMA. B. FACS staining of CFs with Thy1.2 and PDGFRα. C. Phase contrast of CF cultures at d13 of reprogramming in the presence of DMSO or SB431542. Scale bars indicate 100 μm. D. Immunostain of differentiated colony at d21 post‐reprogramming for endothelial and cardiomyocyte markers. E. Schematic detailing lineage tracing experiments utilizing Pdgfra‐CreER‐ROSA26RCAG‐LSL‐EGFP mouse strain and Nkx2‐5 enhancer activity assay using ROSA26RNKX2‐5‐ENHANCER‐BP‐EGFP strain. F. Immunostain of heart sections prepared from mouse in (E, lineage tracing). G. Flow cytometry analysis of CF isolated from mouse in (E) for PDGFRα expression. H. Immunostain of sorted CF reprogrammed in the presence of DMSO or Alk5 inhibitor for Nkx2‐5. I. Immunostain of sorted CF from (E, lineage tracing) for SM‐MHC. J. Immunostain of progenitors derived from sorted CF cultured under SM differentiation conditions for SM‐MHC. Scale bars indicate 50 μm. K. Number of colonies per well derived from sorted CF reprogrammed in DMSO or Alk5i and percentage of SM‐MHC positive cells obtained from either DMSO or Alk5i derived cells. L. Flow cytometry analysis for EGFP expression in cells that have been isolated from mice (E, enhancer activity) and subjected to reprogramming under DMSO or Alk5 inhibitor. Red indicates non‐fluorescent control, green indicates sample. Figure S2: Validation of Alk5 inhibition‐induced reprogramming protocol. A. Phase contrast images depicting reprogramming process conducted with alternative Alk5 inhibitors AZ12799734 and RepSox. B. ciSMP arising from reprogramming detailed in (A) stained for Nkx2‐5 and VIM. C. Further immunostaining for Isl‐1 and Gata4 in enriched ciSMP arising from (A). D. SM differentiation in the absence of TGF‐β demonstrating low differentiation efficiency. E. SM differentiation of ciSMP isolated via (A) and stained for smooth m [file STEM-37-958-s001.docx]

**Supplemental Data**

**CRISPR-KO screen identifies Dmap1 as a regulator of chemically-induced reprogramming and differentiation of cardiac progenitors.**

Jason S. L. Yu, Giorgia Palano, Cindy Lim, Aldo Moggio, Lauren Drowley, Alleyn T. Plowright, Mohammad Bohlooly-Y, Barry S. Rosen, Emil M. Hansson, Qing-Dong Wang, Kosuke Yusa.

**
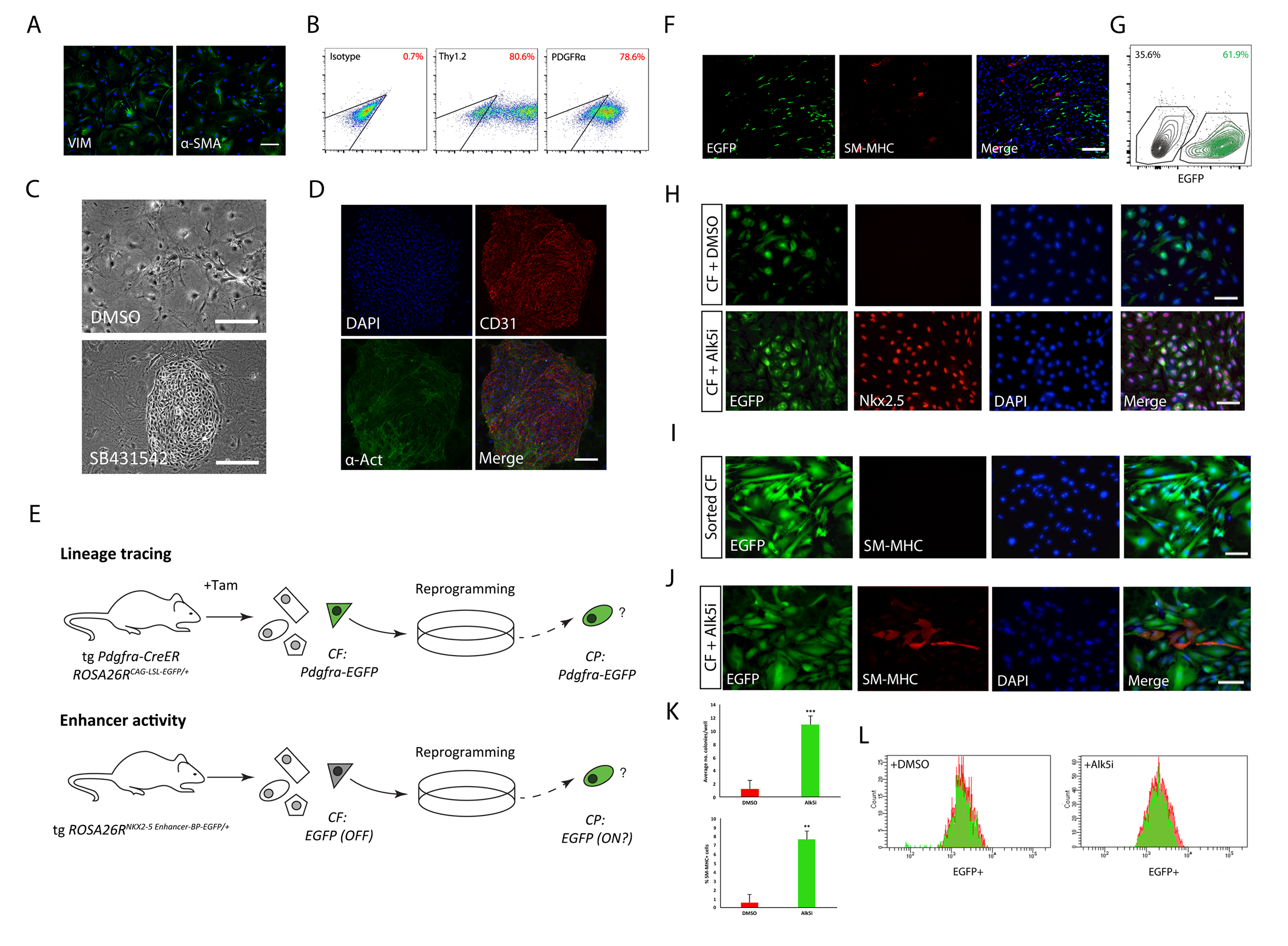
Figure S1: Verification of cardiac fibroblast origin and contribution to reprogramming.**

**A.** Immunostaining in CF for vimentin (VIM) and α-SMA. **B.** FACS staining of CFs with Thy1.2 and PDGFRα. **C.** Phase contrast of CF cultures at d13 of reprogramming in the presence of DMSO or SB431542. Scale bars indicate 100 µm. **D.** Immunostain of differentiated colony at d21 post-reprogramming for endothelial and cardiomyocyte markers. **E.** Schematic detailing lineage tracing experiments utilizing Pdgfra-CreER-ROSA26R^CAG-LSL-EGFP^ mouse strain and Nkx2-5 enhancer activity assay using ROSA26R^NKX2-5-ENHANCER-BP-EGFP^ strain. **F.** Immunostain of heart sections prepared from mouse in (**E**, lineage tracing). **G.** Flow cytometry analysis of CF isolated from mouse in (**E**) for PDGFRα expression. **H.** Immunostain of sorted CF reprogrammed in the presence of DMSO or Alk5 inhibitor for Nkx2-5. **I.** Immunostain of sorted CF from (**E**, lineage tracing) for SM-MHC. **J.** Immunostain of progenitors derived from sorted CF cultured under SM differentiation conditions for SM-MHC. Scale bars indicate 50 µm. **K.** Number of colonies per well derived from sorted CF reprogrammed in DMSO or Alk5i and percentage of SM-MHC positive cells obtained from either DMSO or Alk5i derived cells. **L.** Flow cytometry analysis for EGFP expression in cells that have been isolated from mice (E, enhancer activity) and subjected to reprogramming under DMSO or Alk5 inhibitor. Red indicates non-fluorescent control, green indicates sample.


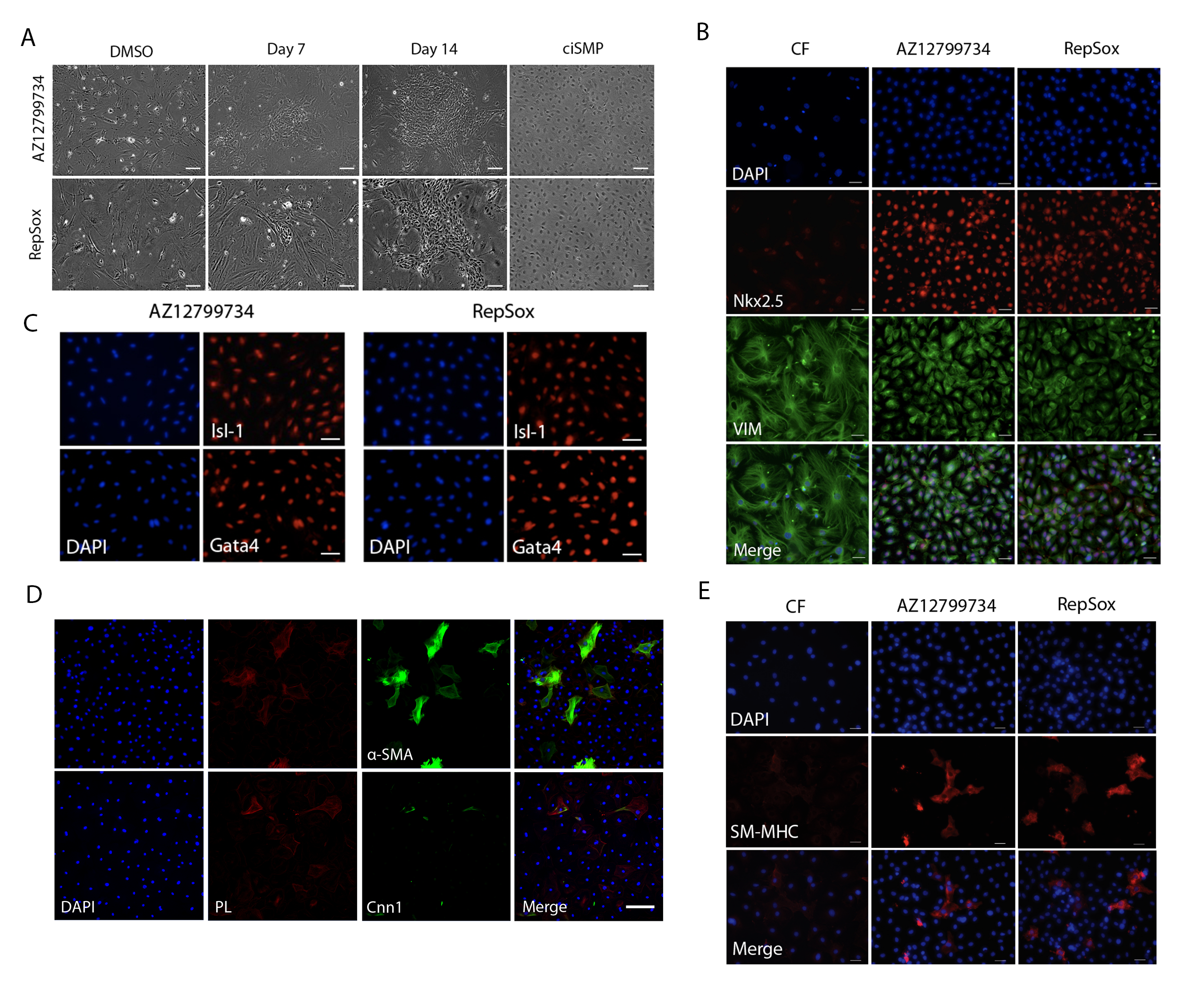


**Figure S2: Validation of Alk5 inhibition-induced reprogramming protocol.**

**A.** Phase contrast images depicting reprogramming process conducted with alternative Alk5 inhibitors AZ12799734 and RepSox. **B.** ciSMP arising from reprogramming detailed in (**A**) stained for Nkx2-5 and VIM. **C.** Further immunostaining for Isl-1 and Gata4 in enriched ciSMP arising from (**A**). **D.** SM differentiation in the absence of TGF-β demonstrating low differentiation efficiency. **E.** SM differentiation of ciSMP isolated via (**A**) and stained for smooth muscle myosin heavy chain (SM-MHC). Scale bars indicates 50 µm.

**
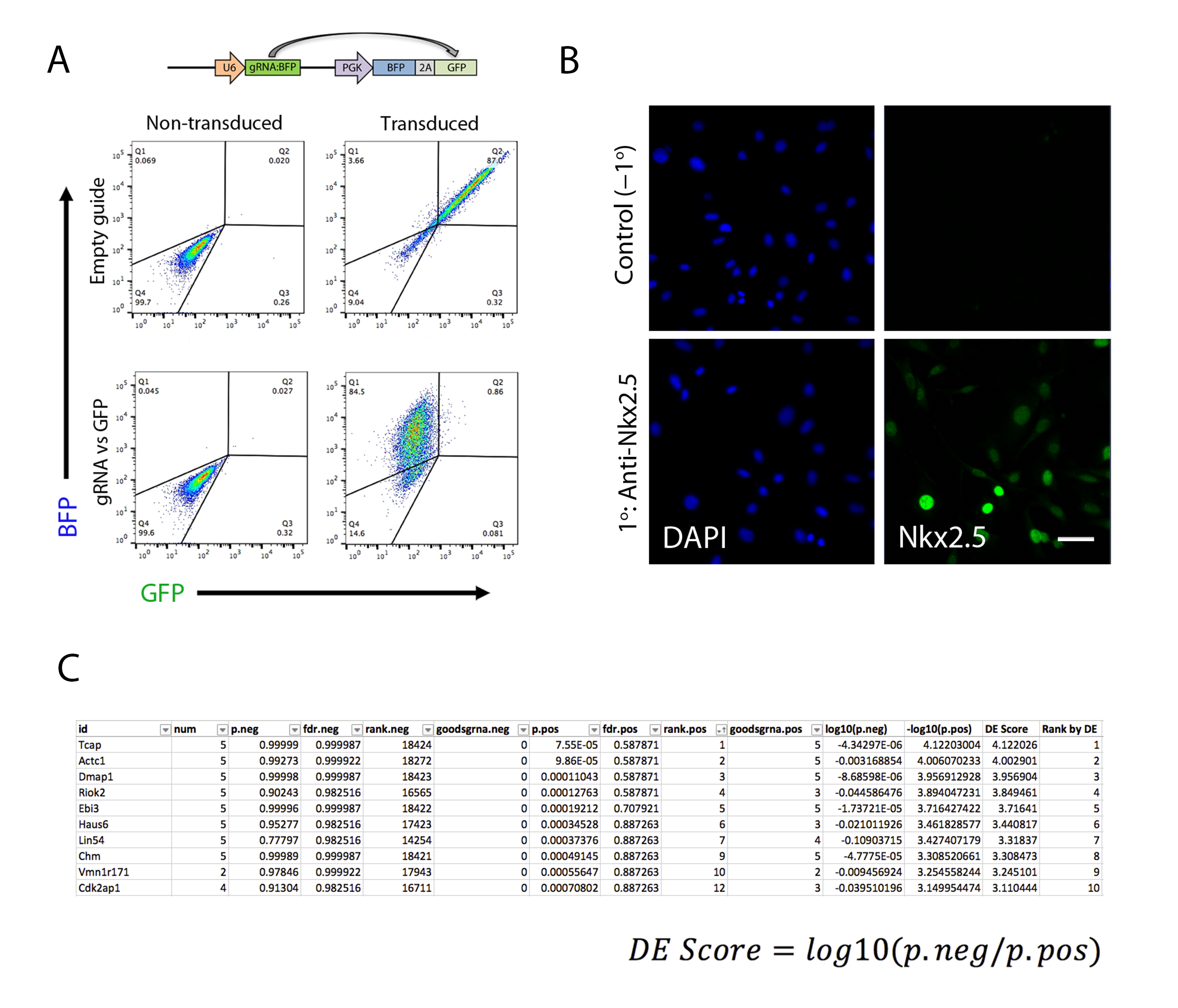
Figure S3: Optimization of CRISPR-KO screen.**

**A.** Assessment of Cas9 activity in CF isolated from Cas9 mice. Cells were transduced with construct containing BFP-2A-GFP with empty guide or self-targeting guide against GFP. Presence and measure of Cas9 activity is indicated by the shift in double positive population to single BFP+ 3 days post transduction. **B.** Testing of antibody specificity in paraformaldehyde-fixed NIH3T3 cells overexpressing Nkx2-5 for use in subsequent FACS. 1^o^ indicates primary antibody. Scale bars indicates 100 µm. **C.** MAGeCK output from gRNA read-counts obtained from next generation sequencing computing abundance of read-counts for a particular gene in Nkx2-5^LOW^ vs Nkx2-5^HIGH^ populations. Id: Gene, num: no. of gRNA targeting gene in library, p.neg/pos: associated P-value for depletion/enrichment, fdr.neg/pos: false discovery rate of hit as depleted/enriched, rank.neg/pos: ranking by MAGeCK based on FDR, goodgrna.neg/pos: no. of gRNA against target gene that based on read counts can be characterised as working, DE score: score calculated as the summation of log10 p.neg and p.pos values where a positive value represents enrichment whilst negative represents depletion relative to Nkx2-5^LOW^ population. DE score was used to rank the genes to produce the hit list and Fig. 2C.

**
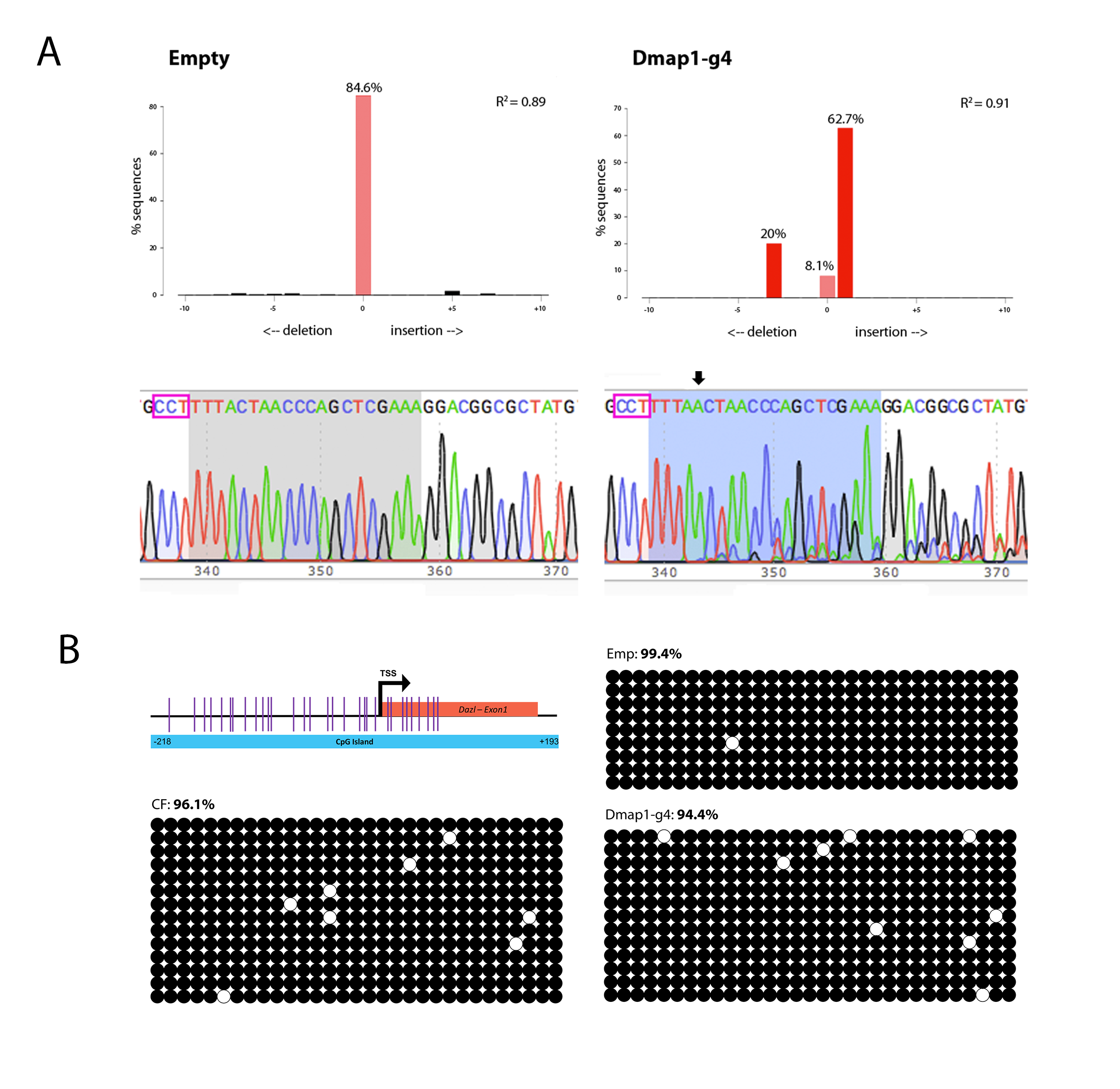
Figure S4: In-depth characterization of Dmap1-KO ciSMP.**

**A.** Tracking of Indels by Decomposition (TIDE) analysis of g4-gRNA mediated editing of the *Dmap1* locus versus unedited empty control. Boxes on respective chromatograms indicate PAM sequence, shaded regions indicate gRNA binding site. Arrow indicates single-base adenine frameshift insertion, the most commonly identified modification in Dmap1-g4 ciSMP (62.7%). **B.** Methylation analysis of the *Dazl* promoter in CF, empty and Dmap1-g4 ciSMPs.

**
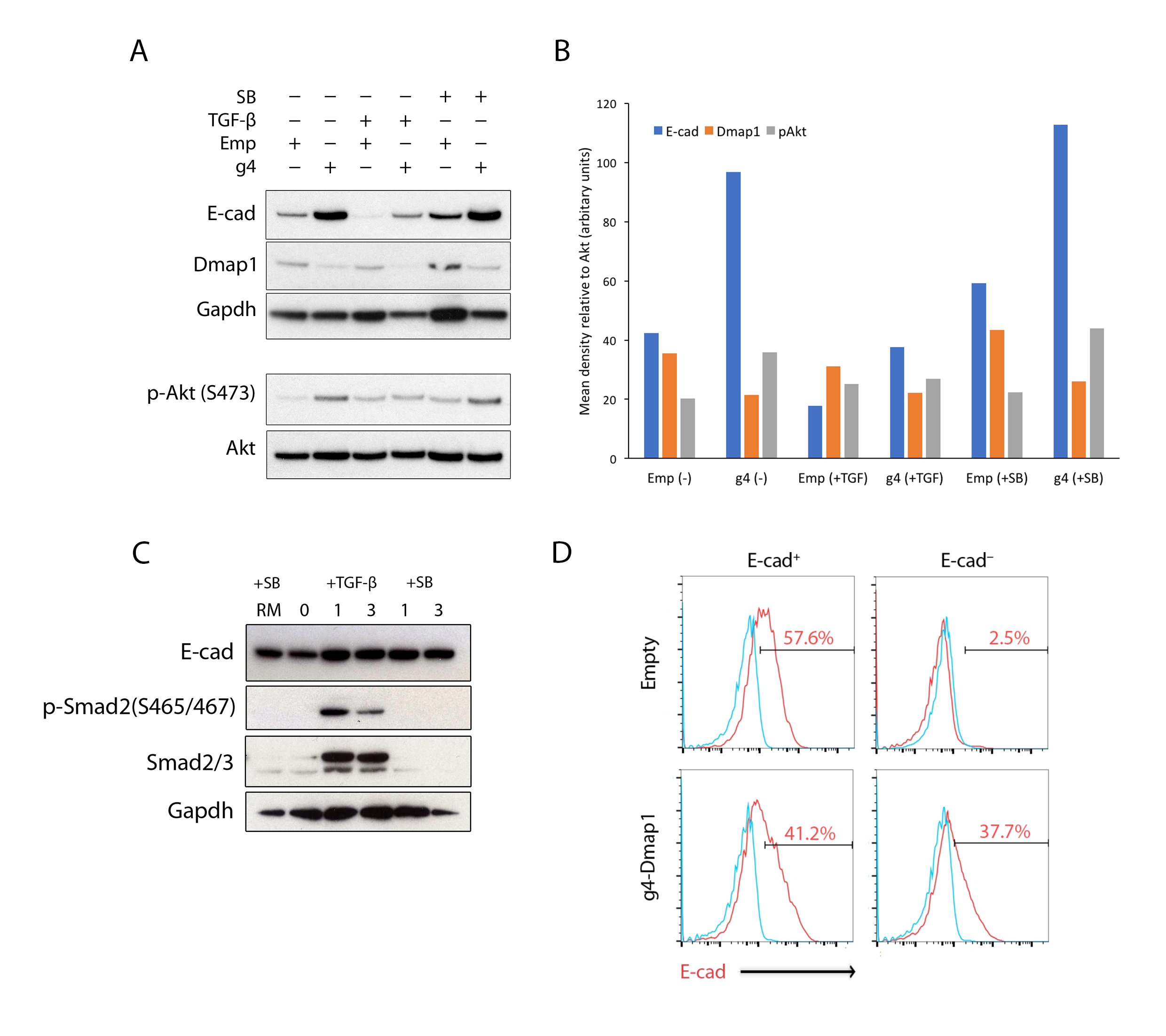
Figure S5: Response of ciSMP to TGF-β signaling during SM differentiation.**

**A.** Immunoblot of ciSMP differentiated overnight with TGF-β or SB for E-cadherin, Dmap1, Gapdh and Akt. Note that transfer of ciSMP into SM inductive conditions significantly alters the expression of Gapdh, however total Akt is unaffected. **B.** Densitometry measurements of (**A**) normalized to total Akt levels. **C.** Acute treatment of g4-ciSMP with TGF-β or SB. RM indicates cells that have been passaged into reprogramming media and left to plate down overnight, whilst ‘0’ time point indicates cells passaged into SM differentiation media without TGF-β or SB. ‘1’ and ‘3’ indicate 1 and 3 h of treatment respectively. **D.** Flow cytometry analysis of E-cadherin expression in control and Dmap1-KO cells that have been passaged twice post-sort.

**Table S1:** Raw read counts and full screen gene list ranked by DE score.

**Table S2:** gRNA used to generate KO lines.

| Gene | gRNA1 |
| --- | --- |
| *Ebi3: 5_1* | GATGCTTCTCGGTATCCCG |
| *Ebi3: 5_3* | GGCCTACAACGGAGCCCCC |
| *Riok2: 5_2* | GCTATCGGTTGACAAATGC |
| *Riok2: 5_3* | GATCGCCATGCAGTGATCA |
| *Dmap1: 5_1 (g1)* | GTTCGTAGTTATTCACGAT |
| *Dmap1: 5_2 (g2)* | GAAGCGCAGATCAAATCGG |
| *Dmap1: 5_3 (g3)* | GATGATGACGCATGGACTA |
| *Dmap1: 5_4 (g4)* | GTCGAGCTGGGTTAGTAAA |
| *Dmap1: 5_5 (g5)* | GTCCAAGAAGGTTCGCCCT |

**Table S3:** Antibodies and dilutions used in this study.

| Antibody target | Supplier/product no. | Application/dilution |
| --- | --- | --- |
| *β-actin* | Sigma: A2228 | IB: 1:20000 |
| *c-Kit* | eBioscience: 10-5805 | FC: 1:100 |
| *Calponin (Cnn1)* | Sigma: C2687 | IF: 1:400 |
| *CD105* | eBioscience: 12-0407 | FC: 1:100 |
| *CD29* | eBioscience: 10-2215 | FC: 1:100 |
| *CD44* | eBioscience: 17-0441 | FC: 1:100 |
| *CD45* | eBioscience: 14-7711 | FC: 1:100 |
| *Dmap1* | Santa Cruz: sc-373949 | IB: 1:500 |
| *E-cadherin (Cdh1)* | BD Bioscience: 610181 | IF: 1:50 |
| *E-cadherin (Cdh1)* | NEB: 3195 | IB: 1:1000 |
| *E-cadherin (Cdh1)* | eBioscience: 14-7303 | FC: 1:100 |
| *Flk1 (KDR)* | eBioscience: 12-5821 | FC: 1:100 |
| *Gapdh* | Millipore: MAB374 | IB: 1:20000 |
| *Gata4* | Santa Cruz: sc-1237 | IF: 1:100 |
| *Gata4* | Thermo Fisher: PA1-102 | IF: 1:100 |
| *GFP* | Abcam: ab13970 | IF: 1:1000 |
| *Irx4* | Abcam: ab123542 | IF: 1:100 |
| *Isl1* | Abcam: ab20670 | IF: 1:100 |
| *Nkx2-5* | Santa Cruz: sc-8697 | IF: 1:50 |
| *Nkx2-5* | R&D Systems: MAB244 | IF: 1:50 |
| *SM-MHC* | Abcam: ab125884 | IF: 1:200 |
| *PDGFRα* | eBioscience: 12-1401 | FC: 1:100 |
| *PECAM (CD31)* | eBioscience: 11-0311 | FC: 1:100 |
| *Sca1* | eBioscience: 11-5981 | FC: 1:100 |
| *α-SMA (Actc2)* | Sigma: A2547 | IF: 1:400 |
| *α-SMA (Actc2)* | Sigma: A5228 | IF: 1:100 |
| *Snai1* | NEB: 3879 | IB: 1:1000 |
| *Tbx5* | Abcam: 137833 | IF: 1:500 |
| *Thy1.2* | eBioscience: 17-0902 | FC: 1:100 |
| *Vimentin (VIM)* | Abcam: ab24525 | IF: 1:200 |
| *Vimentin (VIM)* | Millipore: AB5733 | IF: 1:500 |

**Table S4:** qRT-PCR primers used in this study.

| Gene | Forward | Reverse |
| --- | --- | --- |
| *Actc1* | CTGGATTCTGGCGATGGTGTA | CGGACAATTTCACGTTCAGCA |
| *Acta2* | GTCCCAGACATCAGGGAGTAA | TCGGATACTTCAGCGTCAGGA |
| *Cnn1* | TCTGCACATTTTAACCGAGGTC | GGCTGCATCATTCTTGTCACTT |
| *Dmap1* | GGCGCAGATGTACGAGACATT | GGCCTCTTGAAGGTCAGCG |
| *Ebi3* | CTTACAGGCTCGGTGTGGC | GTGACATTTAGCATGTAGGGCA |
| *Flk1* | TTTGGCAAATACAACCCTTCAGA | GCAGAAGATACTGTCACCACC |
| *Gata4* | CCCTACCCAGCCTACATGG | ACATATCGAGATTGGGGTGTCT |
| *Mef2c* | GTCAGTTGGGAGCTTGCACTA | CGGTCTCTAGGAGGAGAAACA |
| *Myh11* | AAGCTGCGGCTAGAGGTCA | CCCTCCCTTTGATGGCTGAG |
| *Myocd* | GATGGGCTCTCTCCAGATCAG | TCGGATACTTCAGCGTCAGGA |
| *Nkx2-5* | GACAAAGCCGAGACGGATGG | CTGTCGCTTGCACTTGTAGC |
| *Pdgfra* | TCCATGCTAGACTCAGAAGTCA | TCCCGGTGGACACAATTTTTC |
| *Riok2* | TAAGCTGTTCAACAATCCCTCC | GCTGCTTGGTAAACACATTGG |
| *Rpl22* | AGCAGGTTTTGAAGTTCACCC | CAGCTTTCCCATTCACCTTGA |
| *Ryr2* | ACGGCGACCATCCACAAAG | AAAGTCTGTTGCCAAATCCTTCT |
| *Tagln* | CAACAAGGGTCCATCCTACGG | ATCTGGGCGGCCTACATCA |
| *Tbx5* | ATGGCCGATACAGATGAGGG | TTCGTGGAACTTCAGCCACAG |
| *Tcap* | ATGCGCCTGGGTATCCTC | GATCGAGACAGGGTACGGC |
| *Tnni3* | TCTGCCAACTACCGAGCCTAT | CTCTTCTGCCTCTCGTTCCAT |
| *Tnnt2* | CAGAGGAGGCCAACGTAGAAG | CTCCATCGGGGATCTTGGGT |
| *Trp53* | CTCTCCCCCGCAAAAGAAAAA | CGGAACATCTCGAAGCGTTTA |

**Table S5:** Bisulfite cloning primers used in this study.

| Gene | Forward | Reverse |
| --- | --- | --- |
| *Nkx2-5* | TCTCCTTGCAGGAGTGGGA | GATCGAGACAGGGTACGGC |
| *Dazl1* | TAGGGAGTGGTAGGAGTCGGTTTAT | GATAATAAAAAAAAAACCCAC |
| *Cdh1* | TTTAAGGTCGGTTTTATGTTATTAATTATAGATAGGGGTGG | CCTACAACAAAAACAAAAAC |
